# Plant Growth-Promoting Yeasts from *Vitis vinifera* subsp. *sylvestris* as Promising Bioinoculants for Sustainable Crop Production

**DOI:** 10.1021/acs.jafc.5c13229

**Published:** 2026-01-16

**Authors:** María Hernández-Fernández, Gustavo Cordero-Bueso, Jesús Manuel Cantoral

**Affiliations:** Laboratory of Microbiology, Department of Biomedicine, Biotechnology, and Public Health, Faculty of Sciences, 97011 University of Cadiz, 11510 Puerto Real, Cadiz, Spain

**Keywords:** biofertilizers, PGPM, epiphytic yeasts, Vitis sylvestris, Nicotiana tabacum, sustainable agriculture

## Abstract

Plant growth-promoting yeasts are promising bioinoculants for low-input agriculture, yet their application remains underexplored. We isolated 25 epiphytic strains from *Vitis vinifera* subsp. *sylvestris* and performed systematic *in vitro* biochemical profiling of plant growth promoting (PGP) traits. All produced indole-3-acetic acid (IAA); 80% synthesized under L-tryptophan-free conditions, indicating tryptophan-independent routes. ACC deaminase, siderophores, ammonia release, catalase, and biofilm were widespread, whereas nutrient solubilization (P, K, Zn), polyamines, and hydrolases (proteases, chitinases, β-1,3-glucanases, lipases, esterases) were strain-dependent, guiding evidence-based selection. Twelve representatives were evaluated in greenhouse with *Nicotiana tabacum*; ten increased biomass, leaf area, and root traits *versus* the control. The standout strains were *Wickerhamomyces anomalus* C­(H5.1), *Metschnikowia pulcherrima* B­(B5) and C­(A11.2), *Pichia kudriavzevii* C­(A7), and *Yarrowia lipolytica* B­(H3.1.1), each displaying broad functional repertoires and consistent greenhouse performance. Growth promotion occurred without detectable shifts in bulk soil chemistry, supporting native epiphytic yeasts as multifunctional, soil safe bioinoculant candidates.

## Graphical abstract

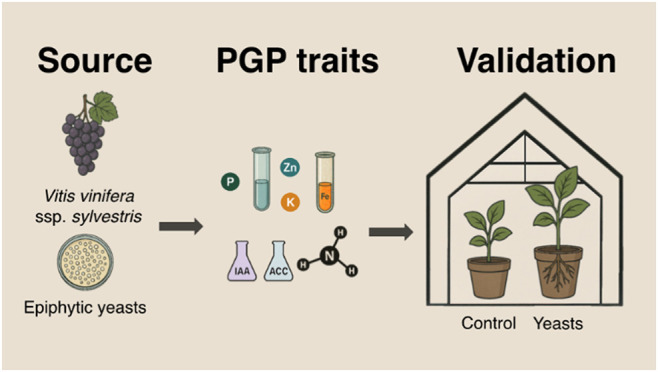

## 1 Introduction

Advancing toward more sustainable, resilient, and environmentally responsible agricultural systems requires alternatives to the intensive reliance on synthetic nutrient inputs. Although mineral fertilization has historically sustained global food production, its overuse has led to serious ecological consequences, including eutrophication, soil acidification, and a decline in microbial biodiversity, ultimately compromising the long-term functionality of agroecosystems.^1^ In this context, plant growth-promoting microorganisms (PGPMs) have gained recognition as biological agents capable of enhancing crop productivity while supporting soil health and agroecological balance.^2,3^

Biofertilizers, microbial formulations containing PGPMs, are designed to stimulate soil biological processes that enhance plant nutrient acquisition through mechanisms such as nitrogen fixation, phosphorus and potassium solubilization, and siderophore-mediated iron mobilization. These inputs typically include live or latent microbial strains that also contribute to phytohormonal regulation and improved plant development.^4,5^ Field trials have shown that their application can increase crop yields by 10–40%, while reducing synthetic input requirements by approximately 25–30%.^6^ Their adoption aligns with agroecological principles and European policy strategies aimed at restoring soil functionality and minimizing chemical dependency.^7^ Reflecting this momentum, the global biofertilizer market has shown sustained expansion, with annual growth rates estimated at around 13% in recent years.^8^ Although field performance remains variable, advances in formulation technologies, microbial consortia, and carrier systems are steadily improving product reliability, efficacy, and scalability.^9^

To date, most commercial products and research efforts have focused on rhizosphere-associated bacteria, such as *Azospirillum*, *Rhizobium*, *Pseudomonas*, and *Bacillus*, along with arbuscular mycorrhizal fungi, all of which enhance plant resilience and nutrient uptake through well-characterized biological strategies.^10,11^ However, these microorganisms often underperform in field conditions due to low environmental persistence, narrow niche specificity, and poor adaptability to abiotic stress.^12^ These limitations have prompted interest in alternative microbial resources with broader ecological plasticity and functional diversity. Some studies indicate that, although yeasts tend to be less abundant than bacteria and filamentous fungi in soil environments, they can persist and remain active under stress, suggesting that their ecological contribution may be underestimated.^13^

In this context, yeasts are emerging as a promising and functionally versatile group of microorganisms with strong potential for biofertilizer development. Although many species are predominantly epiphytic, naturally inhabiting aerial plant organs such as leaves and fruits, they can also persist in soil environments and establish beneficial interactions with plant roots.^14^ Their ecological adaptability and broad spectrum of plant growth-promoting traits include modulation of phytohormonal pathways, enhancement of nutrient availability, mitigation of oxidative and abiotic stress, and improvement of root colonization and plant–microbe interactions.^15,16^ Many yeast strains also support plant health through the production of bioactive compounds and competition against phytopathogens for space and nutrients.^17^ Their ability to withstand harsh environmental conditions, combined with their functional versatility and capacity to interact with host plants through multiple mechanisms, makes them especially attractive for biofertilizer applications in low-input cropping systems.^18^

Several yeast species have been reported to express key PGP traits. For instance, *Metschnikowia pulcherrima* produces indole-3-acetic acid (IAA), siderophores, and hydrolytic enzymes, and suppresses phytopathogens *via* iron competition.^19,20^ *Meyerozyma caribbica* is associated with IAA synthesis and plant growth enhancement, while *Pichia kudriavzevii* exhibits multifunctional activity, including ammonia and siderophore production, nutrient solubilization, and 1-aminocyclopropane-1-carboxylic acid (ACC) deaminase.^21,22^ *Rhodotorula mucilaginosa* also shows similar capabilities.^23^ Other genera such as *Wickerhamomyces*, *Torulaspora*, *Saccharomyces*, *Zygosaccharomyces*, and *Yarrowia* have been linked to nutrient cycling and abiotic stress tolerance.^15,24^

*In planta* studies further support the agricultural potential of yeasts from diverse environments. *Candida tropicalis* rapidly colonized rice roots and increased seedling biomass by up to 35% under greenhouse conditions.^25^ *Meyerozyma guilliermondii* improved seed vigor and reduced fertilizer needs in maize;^26^ *Naganishia uzbekistanensis*, *Papiliotrema terrestris*, and *Solicoccozyma phenolica* supported plant growth under salt stress;^3^ and *Candida guilliermondii* and *R. mucilaginosa* improved lettuce photosynthesis and root development.^27^ Extremophilic yeasts also show PGP traits under harsh conditions.^28^

Despite growing experimental evidence demonstrating their ability to enhance plant growth, nutrient uptake, and stress resilience, yeasts remain notably underrepresented in commercial biofertilizer formulations. While a few experimental or commercial consortia have incorporated yeast strains, most products still prioritize bacterial inoculants or use yeasts mainly for biocontrol.^18,29^ This gap has renewed interest in identifying yeast strains with broader functional capacities and enhanced ecological adaptability. Recent studies suggest that epiphytic yeasts, isolated from various plant species, may offer additional advantages over conventional inoculants by integrating multiple PGP traits within a single organism and exhibiting higher stability under fluctuating environmental conditions.^30–32^

Wild plant epiphytes, particularly those from *Vitis vinifera* subsp. *sylvestris*, offer a valuable microbial reservoir. As the wild ancestor of cultivated grapevine, *V. vinifera* subsp. *sylvestris* thrives in unmanaged ecosystems exposed to nutrient scarcity and recurrent abiotic stress, conditions that likely shape epiphytic microbiota with high functional robustness, ecological plasticity and effective plant–microbe interaction potential.^33–35^ Comparative surveys consistently show that wild grapevines host richer, more heterogeneous and more host-specific microbial communities than domesticated cultivars.^33,36,37^ Agricultural practices such as fungicide use, irrigation and fertilization tend to simplify and homogenize vineyard microbiomes, further widening differences with their wild relatives.^38–41^ Altogether, these findings indicate that wild grapevines act as reservoirs of microbial diversity and functionally relevant traits shaped by natural selection, supporting their relevance as sources of robust epiphytic yeasts for sustainable agriculture.

Building on this ecological background, recent studies show that epiphytic yeasts are not restricted to aerial plant surfaces but can also interact with root-associated environments. Genera commonly found on leaves and fruits, including *Rhodotorula*, *Naganishia*, *Aureobasidium* and *Pseudozyma*, have been shown to promote root development, plant growth and yield when applied to seeds or soil under controlled conditions.^16,30,42^ This belowground performance may be supported by traits selected in epiphytic habitats, including stress tolerance and metabolic versatility. Together, this evidence shows that epiphytic yeasts can function beyond the phyllosphere, supporting their relevance as biofertilizer candidates.

This study reports the isolation and characterization of 25 epiphytic yeast strains from mature berries of *V. vinifera* subsp. *sylvestris*, systematically evaluated for plant growth-promoting potential through *in vitro* assays targeting hormone production, nutrient solubilization, stress tolerance, and biocontrol-related enzymatic activity. Based on these results, a selected subset was tested in greenhouse experiments using *Nicotiana tabacum* as a model plant, due to its sensitivity to phytohormones and frequent use in growth-promotion studies.^43^ Soil physicochemical parameters were also assessed to discern whether the observed effects were driven by rhizosphere-level interactions rather than broad soil modification. This integrative strategy provides novel insights into the biofertilizer potential of wild epiphytic yeasts and supports their application in sustainable agriculture.

## 2 Materials and Methods

### 2.1 Yeast Isolation

Twenty-five epiphytic yeast strains isolated from *Vitis vinifera* subsp. *sylvestris* grape berries collected in El Bosque, Cádiz, Spain (latitude: 0.6416 rad, longitude: −0.0958 rad) in 2022 were evaluated in this study. Ripeness was assessed in the field using a hand-held refractometer (Master Series, ATAGO, Tokyo, Japan), and only berries with °Brix >20 were selected. Approximately 0.50–1.00 kg of fruit was harvested aseptically, transported in coolers, and processed within 3 h at the laboratory. Yeasts were isolated from the berry surface by washing with a saline-Tween 80 solution (9.0 g L^–1^ NaCl, 2.0 g L^–1^ Tween 80), then incubated at 28 °C for 2 h with shaking and brief sonication.^33^ Microorganisms were plated on WL agar (Condalab, Madrid, Spain), incubated at 28 ± 2 °C, and representative colonies were purified on YGC agar (5.0 g L^–1^ yeast extract, 20.0 g L^–1^ glucose, 0.1 g L^–1^ chloramphenicol, 15.0 g L^–1^ agar). Pure cultures were stored at – 80 °C in YPD broth (10 g L^–1^ yeast extract, 20 g L^–1^ peptone, 20 g L^–1^ glucose) supplemented with 20% (v/v) sterilized glycerol. For each assay, overnight YPD broth cultures were pelleted (3000*g*, 5 min, 4 °C), washed twice with sterile saline, and resuspended to an optical density at 600 nm (OD_600_) of 0.10.

### 2.2 Yeast Identification

Yeast identification followed a combined ITS-RFLP and sequencing approach. The ITS1–5.8S-ITS2 region was amplified using primers ITS1/ITS4 under PCR conditions as reported by Hernández-Fernández et al.^44^ Amplicons were digested with HinfI, CfoI, and *Hae*III (Thermo Fisher Scientific, Waltham, MA, USA), following Esteve-Zarzoso et al.^45^ Restriction fragments were resolved on 25 g L^–1^ agarose gels (Condalab, Madrid, Spain) in 1× TBE buffer (89 mM Tris, 89 mM boric acid and 2 mM EDTA, pH 8.3), visualized with ethidium bromide (0.01 g L^–1^, Sigma-Aldrich, St. Louis, MO, USA), and sized using the GeneRuler 100 bp Plus ladder (Thermo Fisher Scientific, Waltham, MA, USA).^46^

Representative amplicons were bidirectionally sequenced by capillary Sanger electrophoresis (EZ-Seq; Macrogen Inc., Seoul, Korea) using ITS1/ITS4 and NL1/NL4 (26S rDNA D1/D2 domain) primers (Kurtzman and Robnett, 1998). Sequences with ≥98% identity and ≥ 90% query coverage in BLAST were assigned to species level and deposited in GenBank.

### 2.3 Quantitative Assays of Growth-Promoting Biomolecules

Yeast cultures were prepared and preadjusted as described in Section 2.1. All assays were performed in triplicate.

#### 2.3.1 IAA Production

Yeast cultures were grown in 5 mL tubes containing YPD broth, with or without 1 g L^–1^ l-tryptophan (l-Trp; Sigma-Aldrich, St. Louis, MO, USA), at 28 ± 2 °C in darkness for 7 d with shaking (150 rpm).^47^ Aliquots collected on days 3, 5, and 7 ^48^ were centrifuged at 6200*g* for 5 min, and supernatants were mixed 1:1 with Salkowski reagent (20 g L^–1^ FeCl_3_·6 H_2_O in 7.9 M H_2_SO_4_) in 96-well plates according to Glickmann and Dessaux.^49^ After 30 min at room temperature in the dark, absorbance was measured at 540 nm using a MultiSkan FC plate reader (Thermo Fisher Scientific, Waltham, MA, USA). IAA concentrations were determined using a standard curve (0–100 μg mL^–1^) prepared with commercial IAA (Glentham Life Sciences, Corsham, U.K.) and expressed as μg mL^–1^. The highest value per strain was recorded.

#### 2.3.2 ACC Deaminase Activity

ACC deaminase activity was assessed following Nutaratat et al.^31^ Yeast suspensions were inoculated into 5 mL of nitrogen-free YCB broth (Difco, Becton Dickinson, Franklin Lakes, NJ, USA) supplemented with 3 mM ACC (Fluorochem, Hadfield, U.K.; filter-sterilized), alongside strain-specific controls without ACC. Cultures were incubated at 28 ± 2 °C for 14 d at 180 rpm. The increase in OD_600_ (day 14–day 0) was calculated for each condition, and net ACC-dependent growth was determined by subtracting the OD_600_ increase in control cultures.

#### 2.3.3 Ammonia (NH_3_) Production

NH_3_ production was evaluated according to the protocol of Cappuccino and Sherman,^50^ with adaptations for microplate-based quantification. Yeast suspensions (100 μL) were inoculated into 5 mL of peptone water (10 g L^–1^ meat peptone, 5 g L^–1^ NaCl; pH 6.8) and incubated at 28 ± 2 °C for 5 d at 180 rpm. After centrifugation (10,000*g*, 5 min), supernatants were mixed (180 μL) with Nessler reagent (20 μL; Supelco, Sigma-Aldrich, St. Louis, MO, USA) in 96-well plates, and absorbance was measured at 450 nm using a MultiSkan FC plate reader (Thermo Fisher Scientific, Waltham, MA, USA). NH_3_ production was quantified using a calibration curve prepared with ammonium sulfate (PanReac AppliChem, Darmstadt, Germany), expressed in mM.

#### 2.3.4 Siderophore Production

Siderophore production was quantified using a modified CAS-shuttle microplate assay.^51^ Adjusted cultures were grown in 5 mL of YPD broth for 48 h (28 ± 2 °C, 180 rpm), centrifuged (10,000*g*, 10 min), and supernatants (100 μL) were mixed 1:1 with liquid CAS reagent (prepared according to Pérez-Miranda et al.,^52^ without agarose) in 96-well plates. After 30 min at room temperature, absorbance was measured at 630 nm using a MultiSkan FC plate reader (Thermo Fisher Scientific, Waltham, MA, USA). Siderophore production was expressed as percent siderophore units (PSU) using the formula

$$\mathrm{PSU}\;\left(\%\right)=\left(\frac{A_{\mathrm{ref}}-A_{\mathrm{sample}}}{A_{\mathrm{ref}}}\right)\times 100$$

where *A*_ref_ is the absorbance of the uninoculated YPD control and *A*_sample_ that of the test sample.

### 2.4 Qualitative Screening of Plant-Beneficial Traits

All assays were performed in triplicate using OD_600_-adjusted cultures as described in Section 2.1. Polyamine, catalase, and HCN activities were visually assessed and classified as low (+), moderate (++), or high (+++) based on reaction intensity. Absence of activity was recorded as (−).

#### 2.4.1 Polyamine Production

Polyamine production was evaluated by spot-inoculating 5 μL of yeast suspensions onto Long Ashton decarboxylase (LAD) agar as described by Cloete et al.,^53^ including parallel control plates without l-arginine. Yeast cultures were incubated at 28 ± 2 °C for 5 d in darkness. Arginine decarboxylase activity, indicative of polyamine production, was determined by the development of red halos around colonies against a yellow background.

#### 2.4.2 Catalase Activity

Catalase activity was tested by adding 5 μL of 3% H_2_O_2_ to colonies on a glass slide. Oxygen bubble formation was observed as an indicator of catalase activity.^30^

#### 2.4.3 Hydrogen Cyanide (HCN) Production

HCN production was assessed by spot-inoculating 5 μL of yeast suspension onto YPD agar supplemented with 4.4 g/L glycine. A picric acid–Na_2_CO_3_–soaked filter paper was attached to the lid, following Nutaratat et al.^31^ A yellow-to-brown color change after 7 d at 28 ± 2 °C indicated HCN production.

#### 2.4.4 Biofilm Formation

Biofilm formation was evaluated following the method of Stepanović et al.,^54^ with modifications. Aliquots (5 μL) were inoculated into 195 μL of YPD broth in 96-well microtiter plates and incubated at 28 ± 2 °C for 18 h. Wells were washed twice with sterile distilled water, stained with 150 μL of 1% crystal violet for 30 min, and washed again. The bound dye was solubilized with 150 μL of 33% acetic acid, and absorbance was measured at 570 nm using a MultiSkan FC plate reader (Thermo Fisher Scientific, Waltham, MA, USA). Uninoculated YPD broth served as a negative control. All experiments were performed in triplicate.

Strains were classified as nonadherent (−), weakly adherent (+), moderately adherent (++), or strongly adherent (+++), based on their OD values. The cutoff OD (OD_c_) was calculated as three standard deviations above the mean OD of the negative control. According to this threshold, strains were categorized as nonadherent (OD ≤ OD_c_), weakly adherent (OD_c_ < OD ≤ 2 × OD_c_), moderately adherent (2 × OD_c_ < OD ≤ 4 × OD_c_), or strongly adherent (4 × OD_c_ < OD).

### 2.5 Extracellular Enzymatic Activity Assays

The production of extracellular hydrolytic enzymes was evaluated by spot-inoculating 5 μL of OD_600_-adjusted yeast suspensions onto specific agar media, followed by incubation at 28 ± 2 °C based on the methods of Fernandez-San Millan et al.,^55^ Hankin and Anagnostakis,^56^ and Sierra,^57^ with minor modifications. Enzymatic activity was assessed by the formation of halos (Chitinase, β-1,3-glucanase, protease, cellulase, pectinase, amylase) or precipitation zones (esterase and lipase), and expressed as degradation activity (DA), calculated as the ratio of the total diameter (colony plus halo or precipitation zone) to the colony diameter. All assays were performed in triplicate.

#### 2.5.1 Chitinase and β-1,3-Glucanase

Chitinase and β-1,3-glucanase activities were screened on modified YPD agar, supplemented with either colloidal chitin (5.0 g L^–1^; Sigma-Aldrich, St. Louis, MO, USA) or yeast β-glucan (2.0 g L^–1^; Biosynth, Staad, Switzerland) at pH 5.5. Colloidal chitin was prepared according to Wen et al.^58^ After incubation for 3 d, plates were stained with 0.2% Congo Red and halos were revealed by rinsing with 1 M NaCl.

#### 2.5.2 Protease

Protease activity was assessed on YMA supplemented with 3% skim milk powder (Condalab, Madrid, Spain; pH 6.8), with halo formation observed after 5 d.

#### 2.5.3 Cellulase, Pectinase, and Amylase

Cellulase and pectinase activities were evaluated on CMC (PanReac AppliChem, Darmstadt, Germany) or citrus pectin (Sigma-Aldrich, St. Louis, MO, USA)-based media, respectively, both adjusted to pH 5.5. Amylase activity was assessed on TSA (Condalab, Madrid, Spain) supplemented with 1% soluble starch (Alfa Aesar, Haverhill, MA, USA). After 5 d of incubation, halos were revealed by flooding the plates with 1% Gram’s iodine solution.

#### 2.5.4 Esterase and Lipase

Esterase and lipase activities were tested on basal agar (10.0 g L^–1^ peptone, 5.0 g L^–1^ NaCl, 0.17 g L^–1^ CaCl_2_·2 H_2_O, 15.0 g L^–1^ agar; pH 7.4) supplemented postautoclaving with 1% (v/v) Tween 80 or Tween 20, respectively. After 5 d of incubation, activity was indicated by the presence of opaque precipitation zones.

### 2.6 Nutrient Solubilization Assays: Phosphate, Zinc Oxide, and Potassium

To assess the solubilization of phosphate, zinc, and potassium, 5 μL of OD_600_-adjusted yeast suspensions were spot-inoculated onto Pikovskaya’s agar^59^ for phosphate, ZnO-modified Pikovskaya’s agar^60^ for zinc, and Aleksandrov agar^61^ for potassium. After 5 d of incubation at 28 ± 2 °C, solubilization efficiency (SE) was calculated as the ratio of the halo diameter to the colony diameter.^55^ All assays were performed in triplicate.

### 2.7 Plant Growth Promotion Assay

Yeast strains were selected for *in planta* assays using a reproducible, trait-based procedure. Strains were (i) grouped by species and ranked using a Functional Trait Score (FTS), defined as the number of positive plant growth-promoting activities; (ii) the highest-scoring isolate of each species was prioritized; (iii) additional isolates were retained only when their FTS values represented distinct performance levels within the same species; and (iv) intermediate-scoring isolates were included when needed to preserve the overall functional gradient of the panel. All decisions followed these FTS-based rules, ensuring that the 12 selected strains reflected the functional breadth of the collection in a consistent and data-driven manner.

#### 2.7.1 Tobacco Seedling Germination

Seeds of *N. tabacum* L. cv. Petite Havana were surface-sterilized in 1% (v/v) sodium hypochlorite and 0.1% (v/v) Tween 20 for 30 min, followed by five rinses with sterile distilled water. Sterilized seeds were placed on Petri dishes containing a solid matrix of deionized water supplemented with 15 g L^–1^ Gelrite (Gelzan CM; Sigma-Aldrich, St. Louis, MO, USA) and sterile filter paper discs moistened with 2 mL of autoclaved tap water. Additionally, 1 mL of sterile distilled water was added on top of the seeds to ensure moisture. Plates were sealed with Parafilm and incubated at 25 ± 2 °C under a 16:8 h light/dark cycle (50 μmol m^–2^ s^–1^) for 30 d.^55^ Uniform seedlings with 3–5 true leaves, comparable size and health, and no visible abnormalities were selected for transplantation.

#### 2.7.2 Tobacco Transplant and Yeast Inoculation

Selected seedlings were transplanted individually into pots (6 cm width × 6 cm depth × 7 cm height) containing 75 g of a sterile 3:1 (v/v) potting soil:vermiculite mixture (COMPO GmbH, Germany; Semillas Batlle S.A., Spain), autoclaved at 120 °C for 20 min. Pots were maintained under greenhouse conditions (16:8 h light–dark cycle, 25 °C, 70–80% relative humidity) with regular and uniform irrigation. After 3 d, 3 mL of OD_600_-adjusted yeast suspension or sterile distilled water (control) were applied to the root zone. Irrigation continued throughout the 50 d experiment. Each strain was tested in three pots, each with three seedlings; pot-level means were used for analysis. The experiment was independently repeated three times.

#### 2.7.3 Plant Growth Evaluation

Plant growth was assessed post-treatment by measuring plant height (from the stem base to the apical point), stem circumference (at the base), number of leaves per plant, maximum leaf area (using ImageJ 1.54g, NIH, Bethesda, MD, USA), total fresh weight (FW), total dry weight (DW) (after oven-drying at 80 °C for 48 h), maximum root length, and chlorophyll (Chl) a + b content (mg g^–1^ FW). Measurements were performed using a measuring tape and an electronic balance, as appropriate. Chl a + b was extracted with 80% acetone and quantified spectrophotometrically at 645 and 663 nm in 96-well microplates using a MultiSkan FC plate reader (Thermo Fisher Scientific, Waltham, MA, USA), following the method of Arnon.^62^

#### 2.7.4 Soil Sampling and Physicochemical Analysis

Separately, fresh substrate was collected at a depth of 2–4 cm for physicochemical characterization, including pH, conductivity, total organic matter (OM), nitrogen (N), calcium (Ca), potassium (K), phosphorus (P), zinc (Zn), sodium (Na), and iron (Fe), which were determined by Cimated Laboratory (Navalmoral de la Mata, Cáceres, Spain). Another aliquot of substrate was processed for DNA extraction (FastDNA Spin Kit for Soil, MP Biomedicals, Irvine, CA, USA) and analyzed by ITS-PCR/RFLP (Section 2.2) to confirm the absence of noninoculated yeast taxa. Root colonization was evaluated following the 50-d assay. Roots were homogenized in PBS–Tween (0.01%), serially diluted, and plated onto WL agar supplemented with chloramphenicol (100 mg L^–1^; Sigma-Aldrich, USA) at 28 ± 2 °C for 48 h. CFU values were recorded, and recovered colonies were identified by ITS-PCR/RFLP (Section 2.2).

### 2.8 Statistical Analysis

Data from quantitative assays were analyzed by one-way analysis of variance (ANOVA) with Tukey’s post hoc test, or by Kruskal–Wallis with Dunn’s post hoc when normality or homoscedasticity assumptions were violated. IAA production (±l-Trp) was compared by paired *t* test.

Plant-growth metrics were subjected to one-way ANOVA with Tukey’s post hoc for all pairwise comparisons and Dunnett’s post hoc against the control. Variables showing high collinearity were removed based on Pearson’s correlations (*r* ≥ 0.9). Multivariate treatment effects were then assessed by MANOVA, followed by pairwise Hotelling’s T^2^ tests on Principal Component Analysis (PCA)–derived component scores (PC1, PC2). Relationships between plant-growth responses and both soil properties and microbial traits were analyzed through a multistep approach. After removing redundant predictors, the remaining variables were evaluated using Spearman’s rank correlations with FDR (false discovery rate) adjustment and visualized in heatmaps. The filtered predictors, including ordinal encodings of qualitative microbial traits, were subsequently scaled and incorporated into exploratory redundancy analysis (RDA) models to examine their combined influence on plant performance. Statistical significance was defined at *p* < 0.05, with *p* < 0.01 noted where applicable. All statistical analyses and graphical visualizations were performed in R (version 4.3.3).

## 3 Results

### 3.1 Yeast Isolation and Molecular Identification

A total of 25 epiphytic yeast isolates from *V. vinifera* subsp. *sylvestris* grape berries were identified to species level based on BLAST comparisons against the GenBank database. Isolates belonged to 13 species across ten genera. *P. kudriavzevii* was the most frequently recovered species, followed by *M. pulcherrima* and *Meyerozyma caribbica*. Full identification details and GenBank accession numbers are provided in Table 1.

**Table 1:** Taxonomic Identification of 25 Wildvine Grapevine Yeast Isolates Based on Sequencing of the ITS and D1/D2 rDNA Regions^a^

		GenBank Acc. No.	
isolate	identification	ITS	D1/D2
B(B5)	*M. pulcherrima*	PV460550	PV564432
C(A11.2)	*M. pulcherrima*	PV545511	PV564442
C(G9)	*M. pulcherrima*	PV545510	PV564441
C(H8.2)	*Metschnikowia viticola*	PV545518	PV564449
A(E2)	*M. caribbica*	PV545521	PV564452
A(F8.2)	*M. caribbica*	PV545509	PV564440
C(E2.1)	*M. caribbica*	PV545504	PV564435
C(A4)	*P. kudriavzevii*	PQ014156	PP990574
C(A7)	*P. kudriavzevii*	PV545513	PV564444
C(B1)	*P. kudriavzevii*	PV545512	PV564443
C(C3.1)	*P. kudriavzevii*	PV545503	PV564434
C(D9)	*P. kudriavzevii*	PV545507	PV564438
C(E5)	*P. kudriavzevii*	PV545517	PV564448
D(F4)	*P. kudriavzevii*	PV545516	PV564447
B(F8)	*Pichia kluyveri*	PQ014155	PP990573
C(F10.4)	*P. kluyveri*	PV545519	PV564450
C(B10)	*Pichia manshurica*	PV545520	PV564451
A(F2.1)	*R. mucilaginosa*	PV545523	PV564454
A(D5)	*Saccharomyces cerevisiae*	PV545508	PV564439
C(B8)	*Torulaspora delbrueckii*	PV545522	PV564453
C(D4.2.1)	*Zygosaccharomyces bailii*	PV545515	PV564446
A(G2)	*Wickerhamomyces anomalus*	PV545505	PV564436
C(H5.1)	*W. anomalus*	PV545506	PV564437
A(E3)	*Yarrowia lipolytica*	PV545514	PV564445
B(H3.1.1)	*Y. lipolytica*	PV545502	PV564433

a GenBank accession numbers (Acc. No.) are indicated for each sequence. Identification was determined by sequence similarity using BLASTn.

### 3.2 Quantitative Assays of Growth-Promoting Biomolecules

All 25 yeast strains were evaluated for their ability to produce IAA, ACC deaminase, ammonium, and siderophores. Results showed high strain-to-strain variability, with no consistent phylogenetic pattern (Figure 1 and Supporting Table S1).

**Figure 1:**
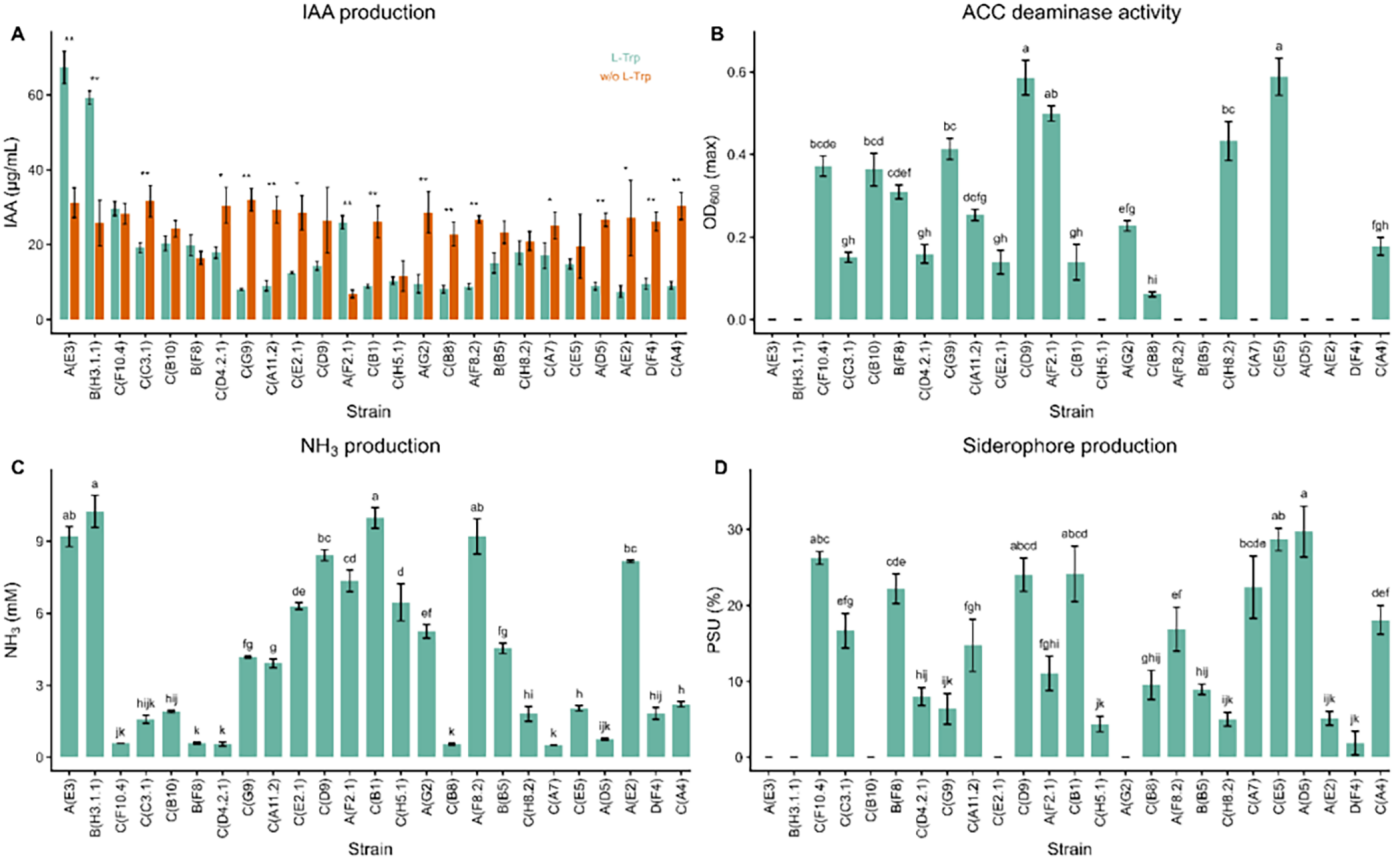
Plant-growth-promoting traits of 25 yeast strains assessed *in vitro*: (A) IAA production with and without (w/o) l-Trp (*p < 0.05; **p < 0.01, paired *t* test), (B) ACC deaminase activity (max. OD_600_), (C) NH_3_ release (mM) and (D) siderophore production (% PSU). Bars represent mean ± SD (*n* = 3); superscript letters denote statistically homogeneous groups (*p* < 0.05, one-way ANOVA with Tukey’s post hoc). Detailed values and groupings are provided in Supporting Table S1.

#### 3.2.1 IAA production

All strains produced IAA under both conditions. Concentrations ranged from 7.52 to 67.30 μg mL^–1^ with l-Trp and from 6.88 to 32.00 μg mL^–1^ without it (Figure 1A and Table S1). Specifically, 80% of strains produced more IAA in the absence of l-Trp. Treatment-related differences were observed in 68% of isolates, and 56% of all strains produced significantly higher levels without supplementation. Only three strains produced more IAA with l-Trp: *Y. lipolytica* strains A­(E3) and B­(H3.1.1), and *R. mucilaginosa* A­(F2.1). The highest values were recorded for A­(E3) and B­(H3.1.1), both differing significantly from the rest, while *M. pulcherrima* C­(G9) was the most productive without l-Trp.

#### 3.2.2 ACC Deaminase Activity

ACC deaminase activity was detected in 64% of the strains, with OD_600_ values ranging from 0.06 to 0.59 (Figure 1B and Table S1). The highest values were recorded for *P. kudriavzevii* strains C­(D9) and C­(E5), both forming the top significance group. *R. mucilaginosa* A­(F2.1) showed similarly high values, though not fully separated from other groups. The lowest activity was observed in *T. delbrueckii* C­(B8).

#### 3.2.3 NH_3_ production

All strains produced NH_3_, with concentrations ranging from 0.50 to 10.24 mM (Figure 1C and Table S1). The highest values were recorded for *Y. lipolytica* B­(H3.1.1) and *P. kudriavzevii* C­(B1), both differing significantly from the other high producers. Several strains, including *T. delbrueckii* C­(B8), *Z. bailii* C­(D4.2.1), *P. kudriavzevii* C­(A7), *P. kluyveri* B­(F8) and *P. kluyveri* C­(F10.4), and *S. cerevisiae* A­(D5), produced less than 1 mM.

#### 3.2.4 Siderophore Production

Siderophore production was detected in 80% of the strains, with values ranging from 1.85 to 29.73% PSU (Figure 1D and Table S1). The highest levels were observed in *S. cerevisiae* A­(D5) and *P. kudriavzevii* C­(E5), though both shared statistical groupings with other high producers. The lowest production among positive strains was recorded for *P. kudriavzevii* D­(F4). No production was detected in five strains, including *Y. lipolytica* strains B­(H3.1.1) and A­(E3), *M. caribbica* C­(E2.1), *W. anomalus* A­(G2), and *P. manshurica* C­(B10).

### 3.3 Qualitative Screening of Plant-Beneficial Traits

#### 3.3.1 Polyamine Production

Polyamine production was detected in 8 strains (32%) (Table 2 and Supporting Figure S1A). Most positive isolates exhibited strong activity (+++). Production was restricted to *P. kudriavzevii* and *W. anomalus*, consistent with a species-specific pattern in this collection.

**Table 2:** Biofilm Formation and Enzyme Activities of 25 Wild Grapevine Yeast Strains Assessed *In Vitro* ^a^

strain	identification	biofilm	polyamine	catalase
C(D9)	*P. kudriavzevii*	**SA (+++)**	**+++**	++
C(E5)		**SA (+++)**	**+++**	++
C(A4)		**SA (+++)**	**+++**	++
C(C3.1)		**SA (+++)**	-	+
C(B1)		**SA (+++)**	**+++**	++
D(F4)		**SA (+++)**	**+++**	++
C(A7)		**SA (+++)**	+	++
B(H3.1.1)	*Y. lipolytica*	MA (++)	-	+
A(E3)		MA (++)	-	++
C(E2.1)	*M. caribbica*	MA (++)	-	+
A(F8.2)		**SA (+++)**	-	+
A(E2)		**SA (+++)**	-	+
C(H5.1)	*W. anomalus*	**SA (+++)**	++	++
A(G2)		MA (++)	**+++**	+
B(F8)	*P. kluyveri*	WA (+)	-	++
C(F10.4)		WA (+)	-	++
C(G9)	*M. pulcherrima*	**SA (+++)**	-	++
C(A11.2)		**SA (+++)**	-	+
B(B5)		**SA (+++)**	-	+
C(D4.2.1)	*Z. bailii*	**SA (+++)**	-	++
C(B10)	*P. manshurica*	**SA (+++)**	-	++
C(H8.2)	*M. viticola*	NA (−)	-	++
C(B8)	*T. delbrueckii*	**SA (+++)**	-	+
A(D5)	*S. cerevisiae*	NA (−)	-	+
A(F2.1)	*R. mucilaginosa*	**SA (+++)**	-	++

a Biofilm adherence was classified according to Stepanović et al.^54^ as strongly adherent (SA, +++), moderately adherent (MA, ++), weakly adherent (WA, +) or not adherent (NA, −). Polyamine and catalase activities were visually scored as low (+), moderate (++) or high (+++); “–” indicates no detectable activity. High-performance values (+++) are shown in bold for clarity.

#### 3.3.2 Catalase Activity

Catalase activity was observed in all strains, with 60% displaying moderate levels (++) and 40% showing low activity (+) (Table 2).

#### 3.3.3 HCN Production

No strain exhibited detectable HCN production under the conditions tested.

#### 3.3.4 Biofilm Formation

All strains could form biofilms except *S. cerevisiae* A­(D5) and *M. viticola* C­(H8.2), which were classified as nonadherent (−) (Table 2). Among the remaining isolates, 68% were strongly adherent (+++), 16% moderately adherent (++), and 8% weakly adherent (+). All *P. kudriavzevii* and *M. pulcherrima* isolates exhibited strong adherence (+++).

### 3.4 Extracellular Enzymatic Activity Assays

The extracellular enzymatic profiles of wild grapevine yeasts were assessed across eight hydrolytic enzymes using DA indices. Among the strains tested, *R. mucilaginosa* A­(F2.1) and *M. caribbica* A­(F8.2) exhibited the broadest activity profiles, each being positive in four different assays. Overall, 64% of the strains showed at least one enzymatic activity.

#### 3.4.1 Chitinase and β-1,3-Glucanase

Chitinase activity was detected in 32% of the strains, while only 16% showed β-1,3-glucanase activity (Table 3 and Supporting Figure S1B,C). *M. caribbica* A­(F8.2) exhibited the highest activity for both enzymes (DA > 2). For Chitinase, *S. cerevisiae* A­(D5) and *P. kluyveri* C­(F10.4) also showed high levels, with significant differences among them.

**Table 3:** *In Vitro* Degradation Activities of 25 Wild Grapevine Yeast Strains^a^

strain	identification	chitinase DA	β-1,3-glucanase DA	protease DA	amylase DA	esterase DA	lipase DA
C(D9)	*P. kudriavzevii*	−	−	−	−	−	−
C(E5)		−	−	−	−	−	−
C(A4)		−	−	−	−	−	−
C(C3.1)		−	−	−	−	−	−
C(B1)		−	−	−	−	−	−
D(F4)		1.41 ± 0.04^ef^	−	−	−	−	−
C(A7)		−	−	−	−	−	−
B(H3.1.1)	*Y. lipolytica*	−	−	1.95 ± 0.13^a^	−	1.20 ± 0.01^c^	−
A(E3)		−	−	1.50 ± 0.09^bc^	−	1.34 ± 0.04^c^	−
C(E2.1)	*M. caribbica*	−	−	−	1.21 ± 0.02^b^		1.36 ± 0.05^d^
A(F8.2)		2.68 ± 0.22^a^	2.00 ± 0.24^a^	−	−	2.10 ± 0.14^b^	1.93 ± 0.14^b^
A(E2)		−	−		−	−	−
C(H5.1)	*W. anomalus*	−	−	1.34 ± 0.18^cd^	−	−	1.48 ± 0.07^d^
A(G2)		−	−	1.64 ± 0.17^b^	−	−	1.71 ± 0.10^c^
B(F8)	*P. kluyveri*	1.63 ± 0.16^d^	−	1.20 ± 0.07^d^	−	−	−
C(F10.4)		1.88 ± 0.08^c^	−	−	−	−	−
C(G9)	*M. pulcherrima*	1.28 ± 0.04^f^	1.37 ± 0.03^b^	1.50 ± 0.05^bc^	−	−	−
C(A11.2)		1.52 ± 0.10^de^	1.30 ± 0.03^b^		−	−	−
B(B5)		1.64 ± 0.06^d^	1.31 ± 0.06^b^	−	−	−	−
C(D4.2.1)	*Z. bailii*	−	−	−	2.08 ± 0.01^a^	−	−
C(B10)	*P. manshurica*	−	−	−	−	−	−
C(H8.2)	*M. viticola*	−	−	−	1.12 ± 0.02^c^	−	−
C(B8)	*T. delbrueckii*	−	−	−	−	−	−
A(D5)	*S. cerevisiae*	2.44 ± 0.07^b^	−	−	−	−	−
A(F2.1)	*R. mucilaginosa*	−	−	1.22 ± 0.07^d^	1.13 ± 0.01^c^	2.56 ± 0.19^a^	2.61 ± 0.09^a^

a Degradation activity (DA) is calculated as (total halo diameter [colony + degradation zone]/colony diameter) in cm; values are mean ± SD (*n* = 3). Superscript letters indicate statistically homogeneous groups (one-way ANOVA, *p* < 0.05, followed by Tukey’s post hoc); “–” indicates no detectable activity.

Chitinase-producing strains were affiliated with *P. kudriavzevii*, *M. caribbica*, *P. kluyveri*, *M. pulcherrima*, and *S. cerevisiae*. β-1,3-glucanase activity was detected only in *M. caribbica* and *M. pulcherrima*, while the remaining strains showed no activity for either enzyme.

#### 3.4.2 Protease

Proteolytic activity was detected in 7 strains (28%) (Table 3 and Supporting Figure S1D), all with DA < 2. *Y. lipolytica* B­(H3.1.1) exhibited the highest activity, significantly higher than the other strains. Other strains, such as *W. anomalus* A­(G2), *Y. lipolytica* A­(E3) and *M. pulcherrima* C­(G9) also showed moderate levels.

#### 3.4.3 Cellulase, Pectinase, and Amylase

None of the tested strains showed detectable cellulase or pectinase activity. In contrast, amylase activity was observed in 16% of the strains (Table 3 and Supporting Figure S1E), with *Z. bailii* C­(D4.2.1) exhibiting significantly the highest activity (DA > 2). Moderate levels were also detected in *M. caribbica* C­(E2.1), *M. viticola* C­(H8.2), and *R. mucilaginosa* A­(F2.1).

#### 3.4.4 Esterase and Lipase

Esterase activity was detected in 16% of the strains, and lipase in 20% (Table 3 and Supporting Figure S1F,G). *R. mucilaginosa* A­(F2.1) showed the highest activity for both enzymes (DA > 2), followed by *M. caribbica* A­(F8.2); both strains differed significantly in each assay. Other esterase-producing strains included *Y. lipolytica*, while lipase activity was also observed in *W. anomalus* strains and *M. caribbica* C­(E2.1), all with moderate levels.

### 3.5 Nutrient Solubilization Assays: Zinc Oxide, Phosphate, and Potassium

Solubilization activity was detected for zinc, phosphate, and potassium in 60%, 36%, and 56% of the strains, respectively (Table 4 and Supporting Figure S1H,I,J). Most positives showed moderate SE values, with a few reaching notably high efficiencies. The highest zinc SE was observed in *P. kudriavzevii* C­(A4), significantly above the rest (SE < 2). For phosphate, the highest SE values (≥2.00) were recorded in *M. pulcherrima* strains C­(G9) and B­(B5). In potassium, *M. pulcherrima* C­(G9) again showed the highest SE = 3.30, followed by *Z. bailii* C­(D4.2.1) and *M. pulcherrima* C­(A11.2), both with SE > 2.50.

**Table 4:** Nutrient-Solubilization Efficiencies of 25 Wild Grapevine Yeast Strains Assessed *In Vitro* ^a^

strain	identification	Zn SE	P SE	K SE
C(D9)	*P. kudriavzevii*	1.45 ± 0.06^acdef^	−	−
C(E5)		1.61 ± 0.06^df^	−	−
C(A4)		1.73 ± 0.08^f^	−	−
C(C3.1)		1.27 ± 0.03^abe^	−	−
C(B1)		1.43 ± 0.16^acde^	−	−
D(F4)		−	1.28 ± 0.04^c^	−
C(A7)		−	−	1.29 ± 0.03^b^
B(H3.1.1)	*Y. lipolytica*	1.57 ± 0.12^df^	−	1.44 ± 0.09^abe^
A(E3)		1.19 ± 0.05^ab^	−	1.72 ± 0.05^acde^
C(E2.1)	*M. caribbica*	−	−	1.45 ± 0.09^abce^
A(F8.2)		1.51 ± 0.15^cdef^	1.27 ± 0.03^c^	1.38 ± 0.05^ab^
A(E2)		−	−	−
C(H5.1)	*W. anomalus*	1.46 ± 0.02^cdf^	1.34 ± 0.07^bc^	
A(G2)		−	−	−
B(F8)	*P. kluyveri*	1.32 ± 0.05^abce^	−	1.82 ± 0.13^acde^
C(F10.4)		−	−	1.44 ± 0.05^ae^
C(G9)	*M. pulcherrima*	1.34 ± 0.07^abce^	2.09 ± 0.12^a^	3.30 ± 0.11^d^
C(A11.2)		1.35 ± 0.08^abce^	1.70 ± 0.01^ab^	2.51 ± 0.10^cde^
B(B5)		1.44 ± 0.11^cde^	2.00 ± 0.22^a^	2.49 ± 0.66^cd^
C(D4.2.1)	*Z. bailii*	−	1.44 ± 0.03^abc^	2.75 ± 0.57^cd^
C(B10)	*P. manshurica*	1.56 ± 0.18^cdf^	−	−
C(H8.2)	*M. viticola*	1.20 ± 0.04^b^	−	1.40 ± 0.06^ab^
C(B8)	*T. delbrueckii*	−	1.49 ± 0.04^abc^	1.38 ± 0.02^ab^
A(D5)	*S. cerevisiae*	−	1.63 ± 0.06^ab^	1.38 ± 0.07^ab^
A(F2.1)	*R. mucilaginosa*	−	−	−

a Zn SE, P SE and K SE indicate zinc, phosphate and potassium solubilization efficiencies, respectively, calculated as (colony + halo diameter)/colony diameter (cm). Values are mean ± SD (n = 3); superscript letters denote statistically homogeneous groups (one-way ANOVA, p < 0.05, Tukey’s post hoc); “–” indicates no detectable activity.

All *M. pulcherrima* strains C­(G9), B­(B5), and C­(A11.2), and *M. caribbica* A­(F8.2) also tested positive in all three assays. In contrast, strains such as *R. mucilaginosa* A­(F2.1) and *M. caribbica* A­(E2) showed no detectable activity. Potassium showed the highest solubilization efficiency and the broadest SE range, from 1.29 to 3.30.

### 3.6 Plant Growth Promotion Assay

Twelve yeast strains were evaluated for their ability to promote tobacco growth under controlled conditions and compared to a noninoculated control. The tested strains included *P. kudriavzevii* C­(D9) and *P. kudriavzevii* C­(A7), *Y. lipolytica* B­(H3.1.1) and *Y. lipolytica* A­(E3), *M. caribbica* A­(F8.2) and *M. caribbica* C­(E2.1), *W. anomalus* C­(H5.1), *P. kluyveri* C­(F10.4), *M. pulcherrima* C­(G9), *M. pulcherrima* B­(B5), and *M. pulcherrima* C­(A11.2), and *R. mucilaginosa* A­(F2.1).

#### 3.6.1 Plant Growth Evaluation

One-way MANOVA on seven morphophysiological traits: plant height, root length, number of leaves, leaf area, stem circumference, Chl a + b content, and FW. DW was excluded due to collinearity with FW (*r* = 0.95). The analysis revealed a highly significant effect of yeast strain (Pillai’s trace = 4.33, *F*_84,175_ = 3.38, *p* < 0.001). PCA yielded two axes (Figure 2). PC1 (eigenvalue = 3.44, 49.14% variance) loaded on aerial biomass (height, stem circumference, FW), leaf area and leaf number. PC2 (eigenvalue = 1.54, 21.99%) contrasting root length with Chl a + b content (cumulative variance 71.13%). Pairwise Hotelling’s T^2^ on PC1–PC2 scores identified ten strains as significantly different from the uninoculated control (*p* < 0.05): *P. kudriavzevii* strains C­(D9) and C­(A7), *Y. lipolytica* B­(H3.1.1), *M. caribbica* C­(E2.1), *W. anomalus* C­(H5.1), *P. kluyveri* C­(F10.4), *M. pulcherrima* strains C­(G9), B­(B5), and C­(A11.2), and *R. mucilaginosa* A­(F2.1). The greatest separation was observed for strain C­(H5.1) (T^2^ = 59.84; df_1_ = 2, df_2_ = 3; p = 0.0038). In contrast, *Y. lipolytica* A­(E3) and *M. caribbica* A­(F8.2) colocalized with the control, indicating no multivariate difference.

**Figure 2:**
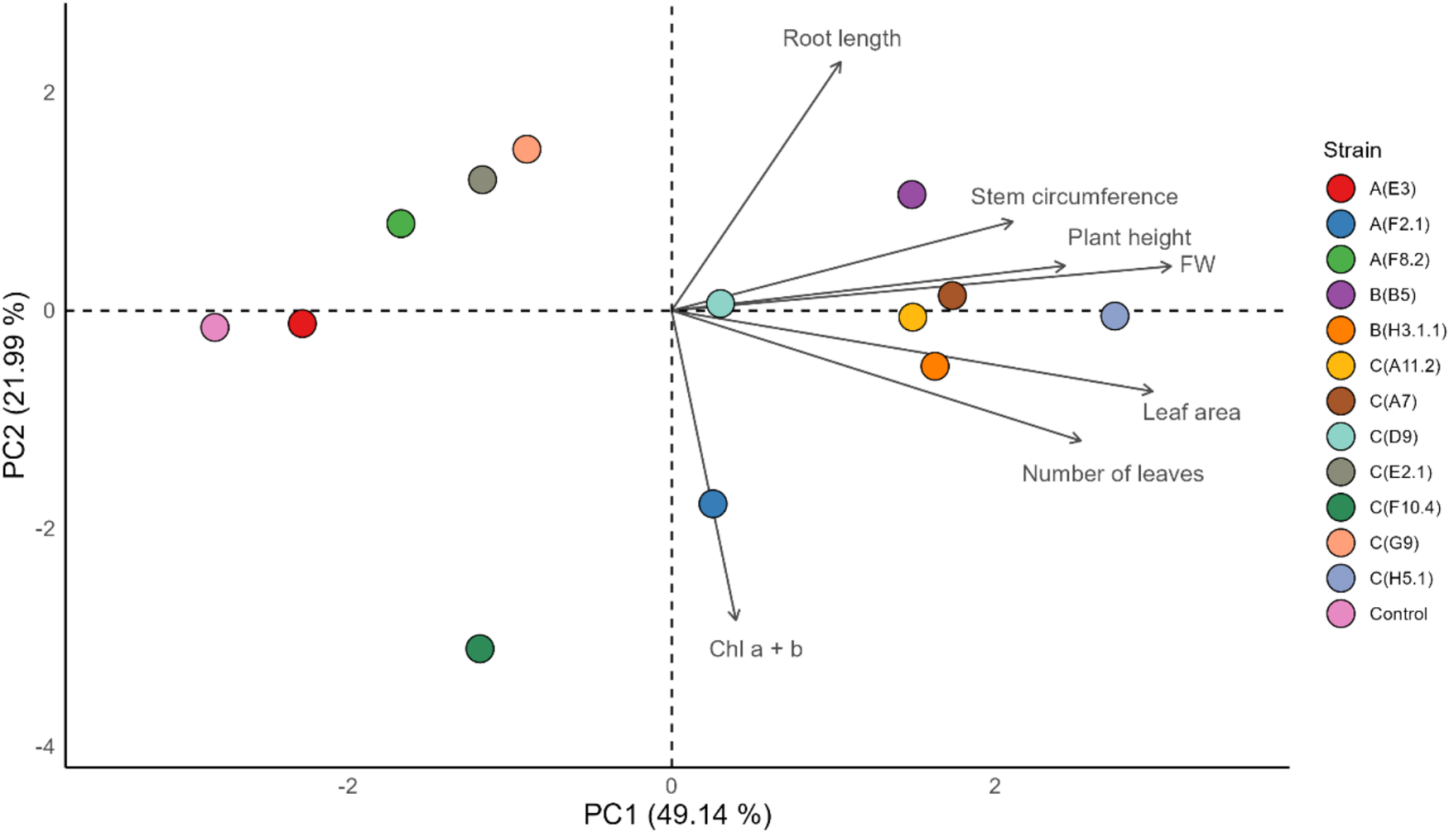
Principal component analysis (PCA) of six morphophysiological traits in tobacco seedlings inoculated with 12 wild grapevine yeast strains and an uninoculated control. Points represent strain centroids (mean ± SD; *n* = 3 pots × 3 seedlings) and the control. Vectors indicate trait loadings: PC1 (49.14% variance) associates with aerial biomass (plant height, stem circumference, fresh weight [FW]), leaf area and leaf number, whereas PC2 (21.99%) contrasts root length against chlorophyll a + b content. Strains on the positive side of PC1 show enhanced above-ground growth relative to the control.

Univariate analysis then established a clear hierarchy of trait responsiveness (Figure 3 and Table S2; detailed p-values reported). Biomass accumulation, based on FW and DW, was enhanced in eight and seven strains, respectively, with *Y. lipolytica* B­(H3.1.1) leading both metrics by nearly tripling FW and boosting DW more than 5-fold *versus* the control. Leaf area followed, enhanced in eight strains: *W. anomalus* C­(H5.1) led with a 2.4-fold increase, *R. mucilaginosa* A­(F2.1), *P. kudriavzevii* C­(A7) and *Y. lipolytica* B­(H3.1.1) each achieved 2.2-fold gains, and the remaining four strains showed 1.3- to 1.8-fold improvements. Root length improved in seven strains, by 80% in *M. pulcherrima* C­(G9) and *M. caribbica* C­(E2.1), and by 70% in *M. pulcherrima* B­(B5). Plant height improved in four strains, with a 2-fold increase in *M. pulcherrima* B­(B5) and 1.7-fold in *Y. lipolytica* B­(H3.1.1). Chlorophyll a + b content increased by over 200% in *P. kluyveri* C­(F10.4) and by 160% in *R. mucilaginosa* A­(F2.1). Stem circumference grew by 70% in *W. anomalus* C­(H5.1) and *P. kudriavzevii* C­(A7), and leaf number also increased by 70% under C­(H5.1). Notably, *W. anomalus* C­(H5.1) enhanced seven traits, followed by *M. pulcherrima* B­(B5) and *P. kudriavzevii* C­(A7) with five each, and *Y. lipolytica* B­(H3.1.1) and *M. pulcherrima* C­(A11.2) with four. Conversely, *Y. lipolytica* A­(E3) and *M. caribbica* A­(F8.2) showed no significant univariate effects. Representative whole-plant phenotypes of these five high-performing strains are shown in Figure 4, illustrating their clear visual differences compared to the uninoculated control.

**Figure 3:**
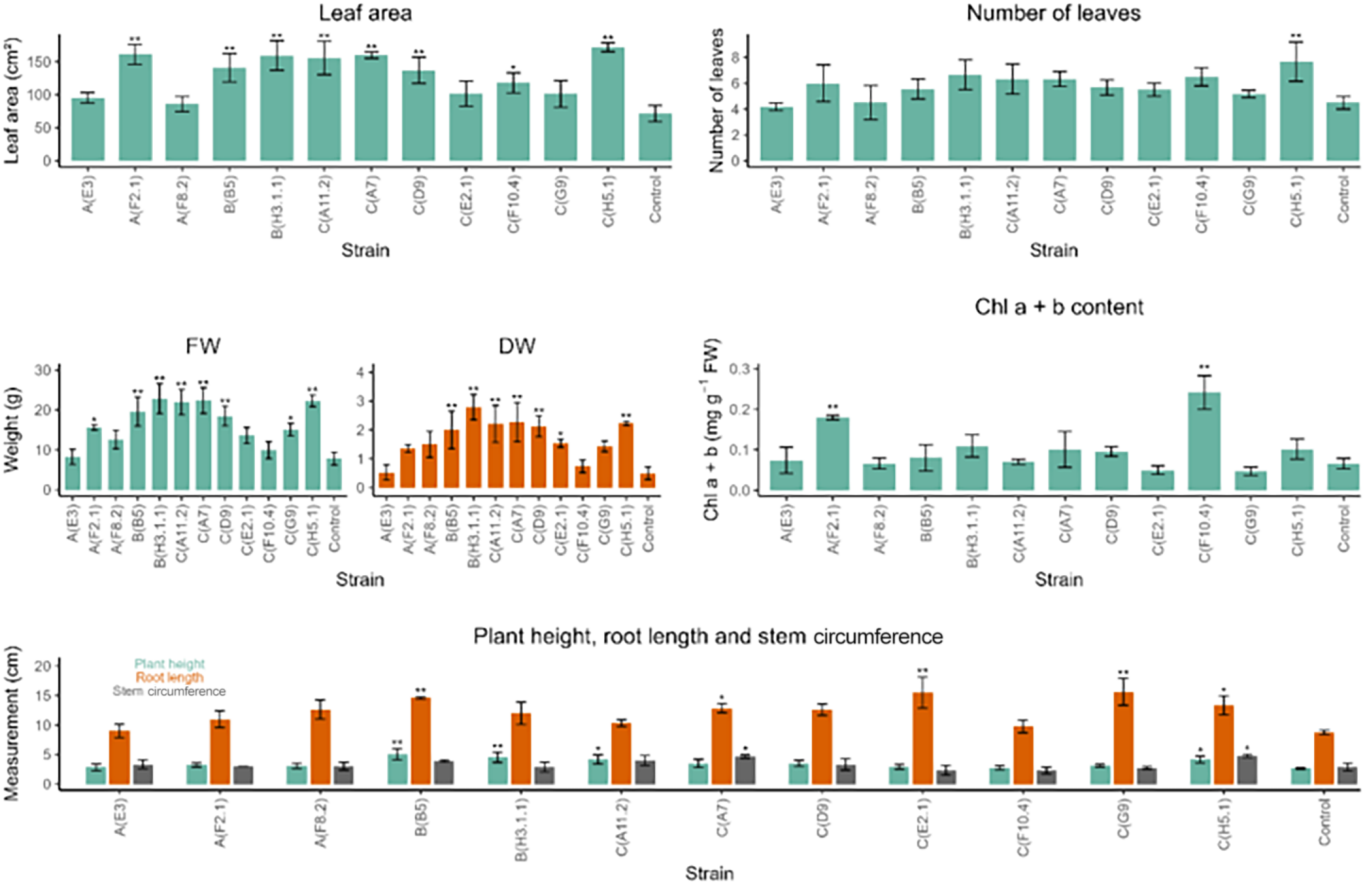
Univariate growth responses of tobacco seedlings inoculated with 12 wild grapevine yeast strains (control = noninoculated). Mean ± SD (*n* = 3 pots × 3 seedlings) for leaf area (cm^2^); number of leaves; fresh weight (FW, g); dry weight (DW, g); chlorophyll (Chl) a + b content (mg g^–1^ FW); and plant height, root length and stem circumference (cm). Asterisks indicate significant differences *versus* control by Dunnett’s test (*p < 0.05; **p < 0.01); full statistical details are in Supporting Table S2.

**Figure 4:**
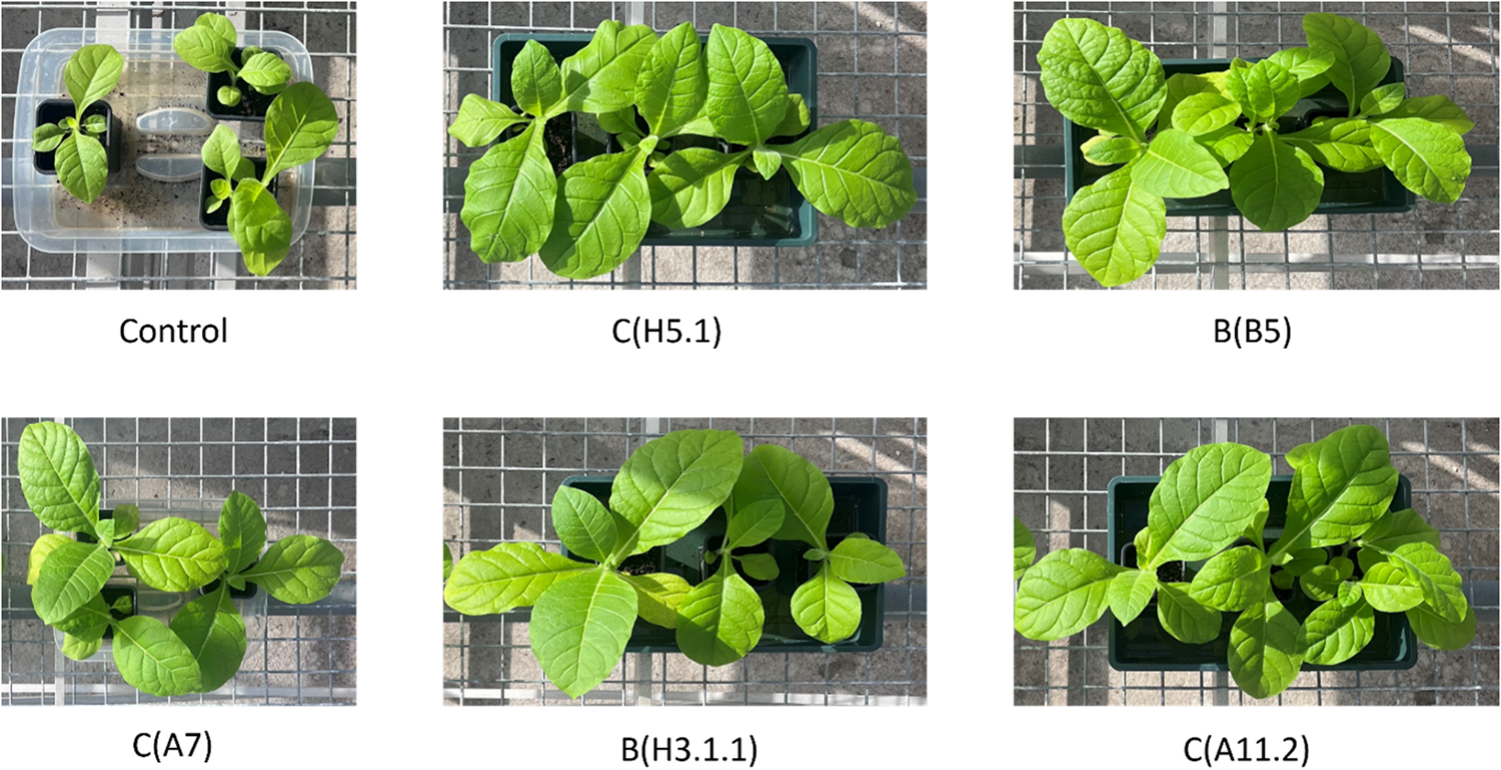
Representative phenotypes of tobacco plants (*Nicotiana tabacum* L. cv. Petite Havana) 50 d after inoculation with five yeast strains showing the strongest growth-promoting effects. Treatments include *W. anomalus* C­(H5.1), *M. pulcherrima* B­(B5), *M. pulcherrima* C­(A11.2), *P. kudriavzevii* C­(A7), *Y. lipolytica* B­(H3.1.1), and the uninoculated control.

#### 3.6.2 Functional Trait-Growth Associations

The set selected after the analyses for exploratory integration consisted of ten nonredundant traits identified as the most representative predictors. These traits were IAA (+l-Trp), IAA (−l-Trp), lipase, β-1,3-glucanase, Zn solubilization, polyamines, biofilm formation, siderophores, NH_3_ production and ACC deaminase.

The correlation heatmap (Figure 5A) revealed directional trends across the 12 strains. Above-ground growth variables showed similar orientations mainly with biofilm formation, Zn solubilization and, to a lesser extent, polyamines, whereas IAA (±l-Trp), NH_3_, lipase and ACC deaminase displayed weak or heterogeneous associations with plant variables. In contrast, β-1,3-glucanase exhibited mixed patterns, being positively associated with plant height but negatively or inconsistently related to leaf-related traits.

**Figure 5:**
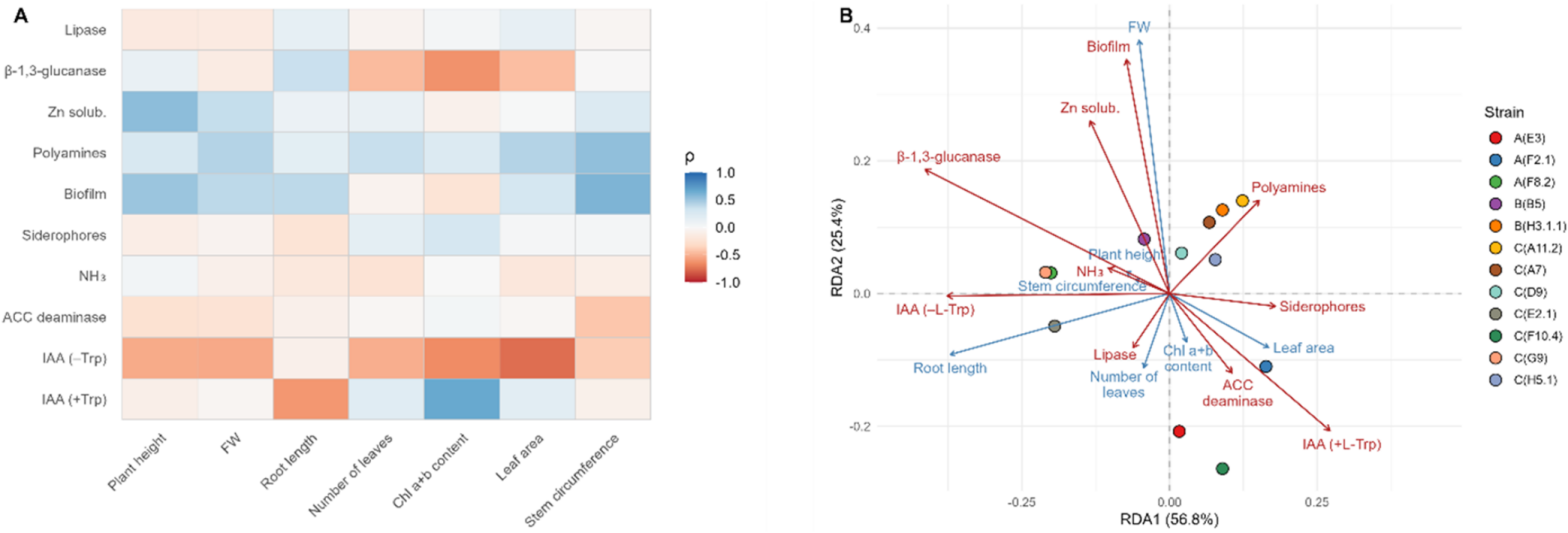
Integration of yeast functional traits with plant-growth responses. (A) Heatmap of Spearman’s ρ between microbial traits and plant-growth variables, with blue for positive and red for negative correlations (intensity ∝ |ρ|). (B) RDA biplot of functional traits (red arrows) and plant variables (blue arrows) on RDA1 (56.80%) and RDA2 (25.40%) of constrained variance; arrows and strain positions reflect the joint distribution of traits and growth responses along the constrained axes.

The RDA (Figure 5B) provided a complementary multivariate representation. RDA1 explained 56.80% of the constrained variation and RDA2 accounted for 25.40%. In the ordination plane, RDA1 grouped variables associated with above-ground growth, while RDA2 represented a secondary gradient contrasting FW and Chl a+b content. Strains that combined several of the traits oriented toward above-ground variables in the heatmap were projected into the region where fresh weight, leaf area, plant height and stem circumference converged. This group included *W. anomalus* C­(H5.1), *M. pulcherrima* B­(B5), *M. pulcherrima* C­(A11.2), *P. kudriavzevii* C­(A7) and *Y. lipolytica* B­(H3.1.1), all of which aligned with the vectors for biofilm formation, Zn solubilization or polyamines.

By contrast, *Y. lipolytica* A­(E3) and *M. caribbica* A­(F8.2) occupied positions away from the main growth-related axes. A­(E3) projected toward lipase, whereas A­(F8.2) oriented toward β-1,3-glucanase and slightly toward root length, indicating that both strains were driven by trait directions different from those associated with plant growth in the ordination.

Intermediate strains appeared in transitional positions, reflecting mixed functional configurations. *M. pulcherrima* C­(G9) combined moderate hydrolytic and nutrient-linked traits; *R. mucilaginosa* A­(F2.1) showed tendencies aligned with foliar variables; and *P. kluyveri* C­(F10.4) and *P. kudriavzevii* C­(D9) expressed subsets of traits associated with individual plant components. C­(G9) and A­(F8.2) occupied neighboring but directionally distinct positions in the ordination, driven by subtle differences in trait orientation, despite partial overlap in some functional tendencies.

#### 3.6.3 Soil Sampling and Physicochemical Analysis

Yeast recovery from root samples following the 50 d assay confirmed that all inoculated strains remained detectable. CFU values fell within the same order of magnitude (10^6^–10^7^ CFU g^–1^), indicating successful establishment under the sterile substrate conditions. Molecular profiling (ITS-PCR/RFLP) verified that only the inoculated yeasts were present in the system.

In the univariate analysis of soil properties, key parameters (pH, conductivity, OM, N, Ca, K, P, Zn, Na and Fe; Table S3) did not differ among treatments (Kruskal–Wallis, *p* > 0.05), nor did a multivariate PERMANOVA on the ten standardized variables reveal any effect (*R* ^2^ = 0.545, *F*_12,13_ = 1.30, p = 0.087).

#### 3.6.4 Soil-Growth Associations

No significant pairwise correlations were detected between any soil parameter and growth metric after FDR adjustment (Spearman’s ρ; all p_adj >0.05; see Supporting Figure S2A), and an exploratory RDA likewise revealed no association between soil chemistry and plant-growth responses (see Supporting Figure S2B).

## 4 Discussion

Global agriculture faces mounting pressure from soil degradation, tighter fertilizer regulations and ambitious environmental targets like the EU’s goal to halve nutrient losses by 2030.^7^ In this scenario, epiphytic yeasts emerge as an underexplored reservoir of PGP potential.^63^ We characterized 25 strains from wild *V. vinifera* subsp. *sylvestris* and selected 12 for greenhouse testing on tobacco, using a standardized commercial substrate. By keeping the growth medium unaltered, we demonstrated consistent growth promotion effects compliant with the efficacy and environmental-safety criteria of Regulation (EU) 2019/1009.^64^ Five isolates, including *W. anomalus* C­(H5.1), *M. pulcherrima* B­(B5) and C­(A11.2), *P. kudriavzevii* C­(A7), and *Y. lipolytica* B­(H3.1.1), consistently enhanced biomass and foliar development, thereby marking them as strong candidates for soil-independent crop improvement. In contrast, *M. caribbica* A­(F8.2) and *Y. lipolytica* A­(E3) had no measurable effect. All isolates were still detectable in the rhizosphere-associated substrate at the end of the trial, indicating that differences in plant responses stemmed from functional traits rather than colonization failure.

To uncover the mechanisms behind these phenotypes, we evaluated key PGP mechanisms. All 25 strains produced IAA, reaching 67.30 μg mL^–1^ with l-Trp and 32.00 μg mL^–1^ without it, implicating biosynthetic routes such as indole-3-pyruvate, indole-3-acetamide, or tryptamine. IAA production in l-Trp-free medium suggests de novo l-Trp biosynthesis *via* the shikimate pathway, although strictly l-Trp-independent mechanisms cannot be excluded.^65^ Similar reports in yeasts show that the extent of endogenous contribution remains unresolved.^18,30,32,55^ Notably, 80% of strains produced more IAA without supplementation, indicating possible feedback repression by exogenous l-Trp. The remaining 20% responded positively to l-Trp, potentially due to limited endogenous synthesis or higher uptake capacity. These responses align with the idea that when exogenous l-Trp is scarce, the l-Trp–IAA pathway remains active through internal synthesis,^66,67^ conferring robustness under nutrient-limited conditions.^32^ However, despite IAA’s central function in regulating root branching, cell elongation and nutrient uptake,^68^ higher IAA levels do not always translate into stronger growth. For instance, *Y. lipolytica* A­(E3) produced the most IAA with l-Trp yet failed to promote root elongation, while *M. pulcherrima* C­(G9), with modest IAA levels, induced the longest roots. Similar discrepancies reported by Carvajal et al.^47^ highlight that auxin synthesis alone is insufficient, and that plant growth responses likely relate to the presence of several microbial traits within each strain rather than to a single mechanism. Consistently, the IAA (±l-Trp) patterns in the heatmap and the orientation of their vectors in the RDA (Figure 5) showed weak correspondence with the main growth-related axes, explaining why A­(E3) positioned outside growth-associated regions despite its high IAA production.

Auxin–ethylene crosstalk and ROS regulation are critical for root development under stress.^69^ IAA induces ACC synthase (ACS), increasing ACC levels, which ACC oxidase (ACO) converts to ethylene in an Fe^2+^-dependent reaction.^70^ Under stress, elevated ROS, particularly H_2_O_2_, amplify ACS/ACO activity, exacerbating growth inhibition.^71^ Microorganisms counter these effects through catalase, which degrades H_2_O_2_;^72^ ACC deaminase, which removes ACC;^69^ siderophore-mediated iron modulation, which restricts ACO activity under Fe^3+^ limitation;^24,73,74^ NH_4_^+^-mediated suppression of ACS induction;^75^ and polyamines, which buffer ROS, stabilize membranes, and modulate ethylene biosynthesis.^76,77^ Since basal ethylene supports root branching and cell expansion whereas excess ethylene inhibits elongation,^69,78^ the modulation of ethylene homeostasis emerges from the combined action of multiple microbial traits.

In our study, 64% of yeast strains exhibited ACC deaminase activity, but only *M. pulcherrima* C­(A11.2) was positive among the top performers. ACC deaminase showed only a slight orientation toward leaf traits in the RDA. Although C­(A11.2) was the only top strain with this activity, it remained grouped with the other high performers, indicating that ACC deaminase did not meaningfully influence the main growth pattern. Nonpromoting strains *Y. lipolytica* A­(E3) and *M. caribbica* A­(F8.2) were also negative for ACC activity, and their RDA projections toward lipase in A­(E3) or β-1,3-glucanase in A­(F8.2) reflected orientations with weak correspondence to plant variables (Figure 5). All strains secreted NH_3_, with the highest levels in *Y. lipolytica* B­(H3.1.1) and A­(E3), followed by *P. kudriavzevii* C­(B1) and *M. caribbica* A­(F8.2), consistent with previous reports.^24,55,79^ However, NH_3_ showed weak and inconsistent relationships with plant variables in Figure 5, aligning with its known role as a colony-synchronization signal rather than a growth determinant.^30,80^ Catalase activity was universal across strains, consistent with its constitutive expression in facultative-aerobic yeasts,^55,81^ and therefore did not distinguish promoters from nonpromoters.

Polyamine production was exclusive to *P. kudriavzevii* and *W. anomalus* isolates. While this trait is well documented in the former,^82^ our study is the first to report it in *W. anomalus*. Inoculation with the polyamine-producing strains *P. kudriavzevii* C­(A7) and *W. anomalus* C­(H5.1) significantly enhanced root elongation. Although this response cannot be attributed solely to polyamines, their roles in oxidative and hormonal regulation suggest they contributed.^83^ Figures 5A–B shows that polyamine production aligned with vegetative traits and projected toward the growth-responsive region of the RDA, converging with the cluster of top performers identified by PCA, indicating that this trait formed part of the multivariate configuration underlying enhanced growth. Siderophore secretion was observed in 80% of isolates, supporting roles in iron acquisition and pathogen inhibition.^84^ Strains of *M. pulcherrima* and *Rhodotorula* consistently produced siderophores,^18^ while *S. cerevisiae* A­(D5) showed the highest production overall, followed by *P. kudriavzevii*.^85,86^ Among growth promoters, *P. kudriavzevii* C­(A7) led in siderophore production, followed by *M. pulcherrima* C­(A11.2) and B­(B5), and *W. anomalus* C­(H5.1), suggesting involvement of iron-related and redox processes. The RDA indicated that siderophores contributed modestly to ordination structure, showing partial alignment with leaf variables, supporting a complementary rather than dominant role. Neither *Y. lipolytica* B­(H3.1.1) nor A­(E3) produced siderophores, whereas *M. caribbica* A­(F8.2) did despite lacking *in planta* effects, highlighting that siderophore secretion is insufficient on its own but can reinforce growth when co-occurring with other relevant traits. None of the strains produced hydrogen cyanide (HCN), consistent with the absence of the *hcnABC* operon in yeast genomes and its restriction to bacterial taxa such as *Pseudomonas* spp.^87^

Biofilm formation, detected in 92% of strains, is a key mechanism in microbial colonization. These structures, composed of exopolysaccharides and adhesins, facilitate surface attachment, nutrient retention, and protection against desiccation and environmental stress.^88,89^ They may also support root colonization and create microniches favorable for seedling establishment.^90^ Certain species, such as *Aureobasidium pullulans*, are known to form protective biofilms with enhanced biocontrol capacity.^91^ Notably, top-performing strains *W. anomalus* C­(H5.1), *M. pulcherrima* B­(B5), *P. kudriavzevii* C­(A7), and *M. pulcherrima* C­(A11.2) formed dense biofilms, while *Y. lipolytica* B­(H3.1.1) showed moderate adhesion. Consistent with their effects on plant development, the RDA placed the biofilm vector toward fresh weight and vegetative traits, and promoters clustered in that direction. In contrast, *Y. lipolytica* A­(E3) and *M. caribbica* A­(F8.2), despite producing substantial biofilms, did not promote growth, and their distant RDA positions indicate that biofilm formation contributes to growth only when combined with other relevant mechanisms.

Yeast colonization also triggered secretion of extracellular hydrolases, involved in nutrient cycling and pathogen suppression. Sixty-four percent of strains produced at least one enzyme. Proteases were particularly relevant, enhancing nitrogen availability and contributing to antifungal activity. *Y. lipolytica* B­(H3.1.1) showed the highest proteolytic index, followed by *W. anomalus* C­(H5.1) and *Y. lipolytica* A­(E3), consistent with reports of antifungal proteases in these species.^92,93^ Chitinases and β-1,3-glucanases, which target fungal cell wall components, were also detected. *M. caribbica* A­(F8.2) showed the strongest activity, in line with its known antifungal potential,^94^ and *S. cerevisiae* A­(D5) showed high Chitinase activity. Among growth-promoting strains, *M. pulcherrima* B­(B5), C­(G9) and C­(A11.2) produced both enzymes, a dual capacity previously linked to postharvest biocontrol.^95^ These activities release N- and C-rich oligomers that enhance microbial competition,^96^ yet only *M. pulcherrima* strains translated them into growth promotion, showing that lytic enzymes alone are insufficient for *in planta* efficacy. Figure 5 showed that β-1,3-glucanase projected mainly along RDA2 and only weakly toward plant-growth vectors: strains projecting toward this vector, such as A­(F8.2), did not promote growth, whereas high producers like B­(B5) and C­(A11.2) appeared on different axes, supporting a limited and context-dependent contribution of this enzyme to the observed growth patterns.

Amylase activity was highest in *Z. bailii* C­(D4.2.1). Esterase and lipase activities were prominent in *R. mucilaginosa* A­(F2.1) and *M. caribbica* A­(F8.2), while *Y. lipolytica* strains B­(H3.1.1) and A­(E3), and *W. anomalus* C­(H5.1) showed selective esterase or lipase production.^97–100^ The RDA indicated that the lipase vector did not align with growth-enhancing traits: strains projecting toward it, such as A­(E3), did not promote growth, whereas lipase-producing promoters like C­(H5.1) occupied different regions of the ordination. All strains were negative for cellulase and pectinase, indicating absence of plant-cell-wall degradation and a low phytotoxicity risk.^101^ Taken together, these results show that extracellular hydrolases contribute to nutrient cycling and antifungal potential but played mainly supportive roles, with only modest correspondence to the main plant-growth axes in the RDA.

Multinutrient solubilization was widespread. Three *M. pulcherrima* strains, C­(G9), B­(B5), and C­(A11.2), mobilized phosphorus, potassium, and zinc, matching previous reports.^55^ *M. caribbica* A­(F8.2) also solubilized all three but did not promote growth, confirming that solubilization alone does not explain plant responses. Figure 5 showed that the Zn-solubilization vector loaded mainly along RDA2 and showed only partial alignment with fresh weight and leaf-related variables, suggesting a moderate but nondominant role in structuring growth outcomes. One-third of strains released phosphate above levels reported in *Drosera*-associated yeasts,^30^ while potassium solubilization was robust, though lower than values reported by Radić et al.^102^ Most *P. kudriavzevii* strains solubilized only zinc,^86^ and *Y. lipolytica* strains B­(H3.1.1) and A­(E3) mobilized both K and Zn. *P. kudriavzevii* C­(A7), despite only solubilizing potassium, was a strong promoter, reinforcing the importance of K mobilization in biofertilizer design.^103^ These results position *M. pulcherrima* as an outstanding multinutrient solubilizer, but ordination analyses indicate that nutrient solubilization acted mainly as a supportive trait that complemented, rather than determined, the multivariate configurations associated with growth promotion.

Based on our *in vitro* findings, greenhouse trials demonstrated that plant growth promotion was not associated with any single trait but rather with the co-occurrence of multiple microbial functions. PCA and MANOVA revealed clear separation between the control and ten inoculated treatments, with strains such as *W. anomalus* C­(H5.1), *M. pulcherrima* B­(B5), *P. kudriavzevii* C­(A7), *Y. lipolytica* B­(H3.1.1), and *M. pulcherrima* C­(A11.2) showing the strongest responses. Consistently, the RDA placed these strains along growth-associated axes, reflecting the combined contribution of multiple traits rather than any single mechanism. *W. anomalus* C­(H5.1) and *M. pulcherrima* B­(B5) exhibited the most comprehensive profiles, driving robust increases in biomass, root length, leaf area, and plant height. *P. kudriavzevii* C­(A7), *Y. lipolytica* B­(H3.1.1), and *M. pulcherrima* C­(A11.2) also performed strongly, each displaying functionally rich profiles with minor trait-specific differences. Despite this variation, all top performers converged along the main growth axes in both PCA and RDA, supporting their consistent growth-promoting behavior. In contrast, *Y. lipolytica* A­(E3) and *M. caribbica* A­(F8.2), which clustered with the control, failed to promote growth despite expressing individual PGP traits; their RDA positions near secondary traits explained their weak association with growth variables.

This was further illustrated by the contrasting performance of two *Y. lipolytica* strains: while B­(H3.1.1) significantly enhanced biomass and leaf area, A­(E3) had no measurable effect, a pattern also reported in other host systems.^55^ Trait-specific outcomes were also evident among top performers: only *M. pulcherrima* strains C­(A11.2) and B­(B5), and *W. anomalus* C­(H5.1) increased plant height; only C­(H5.1) affected leaf number; and only *P. kudriavzevii* C­(A7) and C­(H5.1) expanded stem circumference. Although *P. kluyveri* C­(F10.4) and *R. mucilaginosa* A­(F2.1) increased Chl a + b relative to the control, their modest growth responses confirm that pigment levels alone are not indicative of overall plant performance.^104^ Together, PCA and RDA reinforce that growth promotion arises from multivariate trait combinations, and not from isolated functional capacities. These observations support the potential of epiphytic yeasts as functional inoculants, consistent with previous reports on tobacco.^24^

Crucially, neither Spearman correlations nor redundancy analysis linked plant growth to bulk-soil chemistry, echoing findings by Wang et al.,^105^ who showed that *Bacillus amyloliquefaciens* FH-1 enhanced cucumber performance without altering edaphic parameters. This indicates that growth effects arose independently of substrate chemistry and likely emerged from fine-scale rhizosphere interactions rather than from modifications of the growth medium. Mechanisms likely include root-surface biofilms, siderophore- and polyamine-mediated micronutrient mobilization, enzymatic substrate degradation, and hormonal modulation. Additionally, microbial peptides and volatiles may contribute to systemic plant responses.^106^ To our knowledge, this is the first report showing that wild yeast strains can significantly enhance plant performance without altering bulk soil chemistry.

Although the greenhouse assay relied on a single plant model and a standardized substrate, this design provided the consistency necessary to detect strain-dependent effects. However, determining whether these responses persist under more variable conditions will require evaluation across additional plant hosts, soil types, and abiotic or biotic stresses, including pathogen challenge. *In vitro* PGP profiles offer a functional baseline but do not capture the ecological complexity of soils, where microbial competition and nutrient heterogeneity modulate trait expression.^107,108^ Accordingly, our results represent strain potential under controlled settings and constitute a prerequisite to testing in microbially rich substrates. Assessing performance in nonsterile soils will be particularly relevant to clarify interactions with native communities and strain persistence, ideally through microbiome-based sequencing approaches. Evidence that several yeast species can colonize root-associated habitats and promote growth in nonsterile environments,^109,110^ together with their ability to tolerate microbially dense habitats and form mixed-species biofilms,^14^ supports extending evaluations beyond sterile substrates. Formulation strategies such as alginate encapsulation or coinoculation with compatible rhizobacteria may further enhance viability and root colonization,^111^ and encapsulation has repeatedly been shown to improve yeast survival under demanding conditions and prolong storage stability.^112,113^

While bacterial inoculants still dominate the market, yeasts often withstand desiccation, osmotic stress, and temperature fluctuations more effectively due to their thicker cell walls and epiphytic adaptation.^3,15^ This resilience increases formulation stability and shelf life, frequently surpassing that of many rhizobacteria, which face important storage limitations.^111,114,115^ Advancing these strains toward application will also require deeper molecular characterization: whole-genome sequencing can resolve biosynthetic and regulatory pathways, whereas metabolomics may uncover additional bioactive compounds. Safety assessments are supported by current regulatory designations. *W. anomalus* and *Y. lipolytica* are listed under EFSA QPS,^116^ and *M. pulcherrima* and *P. kudriavzevii* hold GRAS status.^117,118^ Even so, strain-level evaluations remain essential.

Overall, our data indicate that yeast-driven growth promotion results from the combined action of multiple functional traits, consistent with ordination analyses in which growth gradients aligned with multivariate trait configurations rather than isolated mechanisms. Their robustness established safety profiles, and ability to enhance plant performance without altering soil chemistry highlight their potential as next-generation biofertilizers. Consolidating this agronomic value will require validation under heterogeneous, nonsterile, and fluctuating field environments, supported by functional genomics and systems-level approaches.

## Supplementary Material



## Notes

Representative images of qualitative assays for plant growth-promoting traits in epiphytic yeasts; multivariate analyses linking soil physicochemical properties and plant growth responses; comprehensive *in vitro* PGP profiling of 25 yeast strains; greenhouse growth responses of tobacco plants inoculated with selected strains; physicochemical characterization of potting soil supporting the absence of treatment-induced changes (PDF)
