# Supplementary material for: Plant Growth-Promoting Yeasts from Vitis vinifera subsp. sylvestris as Promising Bioinoculants for Sustainable Crop Production
Source: J Agric Food Chem. 2026 Jan 16;74(3):2924–40. doi: 10.1021/acs.jafc.5c13229 (PMC12862773; doi:10.1021/acs.jafc.5c13229)
Supplement: Supplementary file 1 [file jf5c13229_si_001.pdf]

Supporting Information

Plant growth-promoting yeasts from Vitis vinifera subsp. sylvestris as promising bioinoculants for sustainable crop production

María Hernández-Fernández, Gustavo Cordero Bueso, Jesús Manuel Cantoral.

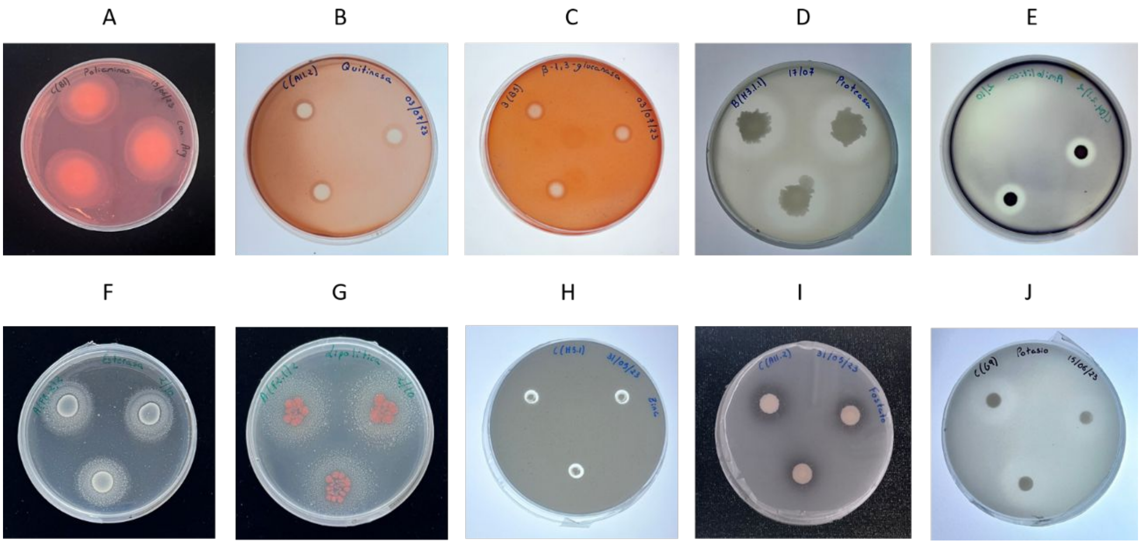

Supplementary Figure S1. Representative images of qualitative assays for plant-beneficial traits in epiphytic yeasts. Assays include: (a) polyamine production (L-arginine medium with Congo red); (b) chitinase activity; (c) β-1,3-glucanase activity (Congo red staining); (d) protease (skim milk agar); (e) amylase (starch hydrolysis); (f) esterase; (g) lipase (Tween-based media); (h) zinc solubilization; (i) phosphate solubilization; and (j) potassium solubilization. Positive activity is indicated by halo formation, precipitation zones, or color changes. Plates were incubated at 28 ± 2 °C for 3–7 days depending on the assay.

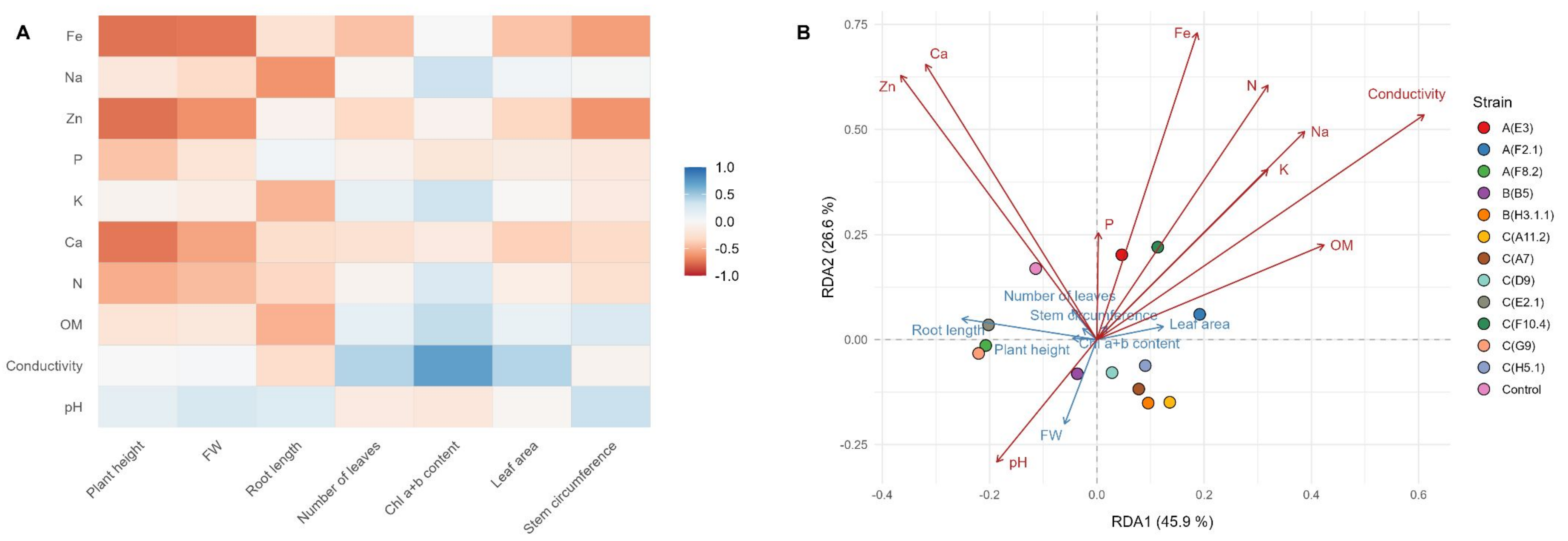

Supplementary Figure S2. Association between soil physicochemical parameters and plant-growth metrics. (A) Heatmap of Spearman's ρ between mean soil properties and plant-growth responses, with blue for positive and red for negative correlations (intensity ∝ │ρ│). (B) RDA biplot of soil variables (red arrows) and plant variables (blue arrows) on RDA1 (45.90%) and RDA2 (26.60%) of constrained variance; arrows are centered near the origin and sample points show no distinct separation along the constrained axes.

Supplementary Table S1. Plant-growth-promoting traits of 25 wild grapevine yeast strains assessed *in vitro*. IAA production with and without Trp (µg mL^{-1}); ACC deaminase production (max. OD_{600}); NH_3 production (µmol mL^{-1}); siderophore production (% PSU). Values are mean ± SD; superscripts: letters indicate statistically homogeneous groups ($p < 0.05$, one-way ANOVA with Tukey's post-hoc test) and asterisks denote differences in IAA production between conditions (paired t-test; *p < 0.05, **p < 0.01); "–" indicates no detectable activity.

Strain	Identification	IAA production (µg mL^{-1})		ACC deaminase production (max. OD_{600})	NH_3 production (mM)	Siderophore production (%)
		with Trp	w/o Trp			
C(D9)	*Pichia kudriavezvii*	14.39 ± 1.15^{efg}	26.51 ± 8.74^{ab}	0.59 ± 0.04^{a}	8.43 ± 0.23^{bc}	24.03 ± 2.18^{abcd}
C(E5)		14.86 ± 1.33^{efg}	19.57 ± 8.55^{abc}	0.59 ± 0.05^{a}	2.04 ± 0.12^{h}	28.68 ± 1.48^{ab}
C(A4)		9.11 ± 1.05^{gh}	30.35 ± 3.63^{a**}	0.18 ± 0.02^{fgh}	2.21 ± 0.11^{h}	18.09 ± 1.89^{def}
C(C3.1)		19.20 ± 1.35^{ef}	31.59 ± 4.19^{a**}	0.15 ± 0.01^{gh}	1.59 ± 0.17^{hijk}	16.66 ± 2.30^{efg}
C(B1)		8.99 ± 0.48^{gh}	26.08 ± 4.31^{ab**}	0.14 ± 0.04^{gh}	9.98 ± 0.43^{a}	24.13 ± 3.65^{abcd}
D(F4)		9.55 ± 1.51^{gh}	26.24 ± 2.52^{ab**}	–	1.83 ± 0.24^{hij}	1.85 ± 1.57^{jk}
C(A7)		17.08 ± 3.39^{ef}	25.12 ± 3.61^{ab*}	–	0.50 ± 0.02^{k}	22.39 ± 4.11^{bcde}
B(H3.1.1)	*Yarrowia lipolytica*	59.25 ± 1.82^{b}	25.78 ± 6.14^{ab**}	–	10.24 ± 0.67^{a}	–
A(E3)		67.30 ± 4.35^{a}	31.21 ± 3.97^{a**}	–	9.19 ± 0.42^{ab}	–
C(E2.1)	*Meyerozyma caribbica*	12.53 ± 0.28^{fgh}	28.51 ± 4.59^{ab*}	0.14 ± 0.03^{gh}	6.30 ± 0.14^{de}	–
A(F8.2)		8.86 ± 0.77^{gh}	26.74 ± 0.99^{ab**}	–	9.20 ± 0.74^{ab}	16.88 ± 2.90^{ef}
A(E2)		7.52 ± 1.54^{h}	27.11 ± 10.10^{ab*}	–	8.17 ± 0.05^{bc}	5.14 ± 0.91^{ijk}
C(H5.1)	*Wickerhamomyces anomalus*	10.39 ± 1.02^{gh}	11.61 ± 4.08^{bc}	–	6.46 ± 0.77^{d}	4.39 ± 1.03^{jk}
A(G2)		9.56 ± 2.43^{gh}	28.67 ± 5.48^{ab**}	0.23 ± 0.01^{efg}	5.24 ± 0.29^{ef}	–

B(F8)	*Pichia kluyveri*	19.87 ± 2.80^{de}	20.39 ± 1.80^{abc}	0.31 ± 0.02^{cdef}	0.59 ± 0.04^{k}	22.18 ± 1.94^{cde}
C(F10.4)		29.63 ± 1.87^{c}	28.31 ± 2.80^{ab}	0.37 ± 0.02^{bcde}	0.58 ± 0.00^{jk}	26.24 ± 0.85^{abc}
C(G9)	*Metschnikowia pulcherrima*	8.09 ± 0.28^{gh}	32.00 ± 3.09^{a**}	0.41 ± 0.03^{bc}	4.19 ± 0.04^{fg}	6.38 ± 2.01^{ijk}
C(A11.2)		9.05 ± 1.38^{gh}	29.36 ± 3.62^{ab**}	0.25 ± 0.01^{defg}	3.92 ± 0.18^{g}	14.75 ± 3.44^{fgh}
B(B5)		15.11 ± 2.67^{efg}	23.33 ± 2.98^{ab}	–	4.54 ± 0.22^{fg}	8.96 ± 0.67^{hij}
C(D4.2.1)	*Zygosaccharomyces bailii*	17.90 ± 1.52^{ef}	30.51 ± 4.77^{a*}	0.16 ± 0.02^{gh}	0.55 ± 0.09^{k}	7.99 ± 1.20^{hij}
C(B10)	*Pichia manshurica*	20.40 ± 1.98^{de}	24.29 ± 2.18^{ab}	0.36 ± 0.04^{bcd}	1.90 ± 0.05^{hij}	–
C(H8.2)	*Metschnikowia viticola*	17.88 ± 3.14^{ef}	20.99 ± 2.52^{abc}	0.43 ± 0.05^{bc}	1.82 ± 0.31^{hi}	5.02 ± 0.90^{ijk}
C(B8)	*Torulaspora delbrueckii*	8.13 ± 1.04^{gh}	22.83 ± 3.19^{ab**}	0.06 ± 0.01^{hi}	0.55 ± 0.04^{k}	9.51 ± 1.93^{ghij}
A(D5)	*Saccharomyces cerevisiae*	8.90 ± 0.98^{gh}	26.69 ± 1.80^{ab**}	–	0.76 ± 0.04^{ijk}	29.73 ± 3.35^{a}
A(F2.1)	*Rhodotorula mucilaginosa*	25.97 ± 1.76^{cd}	6.88 ± 1.03^{c**}	0.50 ± 0.02^{ab}	7.36 ± 0.44^{cd}	11.05 ± 2.24^{fghi}

Supplementary Table S2. Univariate growth responses of tobacco seedlings inoculated with 12 wild grapevine yeast strains and a non-inoculated control. Mean ± SD (n = 3 pots × 3 seedlings) are shown for plant height (cm), root length (cm), number of leaves, stem circumference (cm), leaf area (cm^2), fresh weight (FW, g), dry weight (DW, g) and chlorophyll (Chl) a + b content (mg g^{-1} FW). Superscript letters indicate statistically homogeneous groups (one-way ANOVA, $p < 0.05$, followed by Tukey's post hoc), and asterisks denote significant differences versus control by Dunnett's test (*$p < 0.05$; **$p < 0.01$).

Strain	Identification	Plant height (cm)	Root length (cm)	Number of leaves	Stem circumference (cm)	Leaf area (cm^2)	FW (g)	DW (g)	Chl a + b (mg g^{-1} FW)
C(D9)	*Pichia kudriavezvii*	3.50 ± 0.50^{abc}	12.60 ± 0.95^{abcd}	5.67 ± 0.58^{ab}	3.30 ± 0.96^{abc}	136.92 ± 19.83^{ab**}	18.49 ± 2.46^{abc**}	2.12 ± 0.36^{ab**}	0.10 ± 0.01^{c}
C(A7)		3.50 ± 0.70^{abc}	12.83 ± 0.76^{abcd*}	6.33 ± 0.58^{ab}	4.67 ± 0.29^{ab*}	159.91 ± 4.82^{a**}	22.34 ± 3.19^{ab**}	2.27 ± 0.68^{ab**}	0.10 ± 0.04^{c}
B(H3.1.1)	*Yarrowia lipolytica*	4.50 ± 0.87^{ab**}	12.00 ± 1.90^{abcd}	6.67 ± 1.15^{ab}	2.87 ± 0.81^{abc}	159.30 ± 22.44^{a**}	22.86 ± 3.74^{a**}	2.79 ± 0.44^{a**}	0.11 ± 0.03^{bc}
A(E3)		2.83 ± 0.58^{bc}	9.00 ± 1.15^{cd}	4.17 ± 0.29^{b}	3.33 ± 0.76^{abc}	95.28 ± 8.08^{bc}	8.26 ± 1.88^{d}	0.53 ± 0.26^{d}	0.07 ± 0.03^{c}
C(E2.1)	*Meyerozyma caribbica*	2.92 ± 0.38^{bc}	15.50 ± 2.65^{a**}	5.50 ± 0.50^{ab}	2.37 ± 0.82^{c}	101.61 ± 18.88^{bc}	13.59 ± 2.03^{cd}	1.53 ± 0.14^{bcd*}	0.05 ± 0.01^{c}
A(F8.2)		3.08 ± 0.43^{bc}	12.63 ± 1.65^{abcd}	4.50 ± 1.32^{b}	3.00 ± 0.66^{abc}	86.01 ± 11.43^{c}	12.62 ± 2.31^{cd}	1.50 ± 0.45^{bcd}	0.07 ± 0.01^{c}
C(H5.1)	*Wickerhamomyces anomalus*	4.17 ± 0.58^{abc*}	13.33 ± 1.60^{abc*}	7.67 ± 1.53^{a**}	4.77 ± 0.25^{a*}	171.80 ± 6.65^{a**}	22.30 ± 1.46^{ab**}	2.23 ± 0.07^{ab**}	0.10 ± 0.03^{c}
C(F10.4)	*Pichia kluyveri*	2.75 ± 0.35^{bc}	9.75 ± 1.06^{bcd}	6.50 ± 0.71^{ab}	2.30 ± 0.57^{c}	117.94 ± 15.29^{abc*}	9.94 ± 2.11^{d}	0.74 ± 0.22^{cd}	0.24 ± 0.04^{a**}

C(G9)		3.15 ± 0.25^{bc}	15.62 ± 2.27^{a**}	5.17 ± 0.29^{ab}	2.75 ± 0.25^{c}	101.31 ± 20.21^{bc}	15.08 ± 1.58^{bcd*}	1.43 ± 0.19^{bcd}	0.06 ± 0.01^{c}
C(A11.2)	*Metschnikowia pulcherrima*	4.17 ± 0.55^{abc*}	10.33 ± 0.58^{bcd}	6.33 ± 1.15^{ab}	4.00 ± 0.87^{abc}	155.67 ± 25.48^{a**}	21.95 ± 3.21^{ab**}	2.58 ± 0.10^{ab**}	0.07 ± 0.01^{c}
B(B5)		5.06 ± 0.96^{a**}	14.60± 0.20^{ab**}	5.56 ± 0.77^{ab}	3.89 ± 0.19^{abc}	140.71 ± 21.29^{ab**}	19.59 ± 3.66^{abc**}	2.00 ± 0.65^{abc**}	0.08 ± 0.03^{c}
A(F2.1)	*Rhodotorula mucilaginosa*	3.25 ± 0.35^{abc}	11.00 ± 1.41^{abcd}	6.00 ± 1.41^{ab}	3.00 ± 0.00^{abc}	160.85 ± 15.13^{a**}	15.65 ± 0.63^{abcd*}	1.35 ± 0.13^{bcd}	0.18 ± 0.01^{ab**}
Control	Untreated	2.67 ± 0.14^{c}	8.78 ± 0.40^{d}	4.50 ± 0.50^{b}	2.85 ± 0.65^{bc}	71.43 ± 12.27^{c}	7.82 ± 1.58^{d}	0.50 ± 0.21^{d}	0.07 ± 0.01^{c}

Supplementary Table S3. Physicochemical properties of potting soil from experimental pots inoculated with 12 wild grapevine yeast strains and a non-inoculated control. Mean ± SD (n = 3 soil samples per treatment) are shown for pH (pH units), conductivity (mS cm^{-1}), total organic matter (OM, %), nitrogen (N, %), and concentrations of calcium (Ca), potassium (K), phosphorus (P), zinc (Zn), sodium (Na) and iron (Fe) (all in ppm).

No significant differences were detected among treatments by per-variable Kruskal–Wallis tests (all p > 0.05).

Strain	Identification	pH	Conductivity	OM	N	Ca	K	P	Zn	Na	Fe
C(D9)	*Pichia kudriavezvii*	5.71 ± 0.11	1.46 ± 0.05	61.51 ± 9.38	0.96 ± 0.04	9,437.84 ± 2,509.58	203.32 ± 160.36	49.63 ± 14.67	11.65 ± 0.13	571.55 ± 187.03	63.93 ± 3.95
C(A7)		5.98 ± 0.37	1.39 ± 0.29	50.71 ± 2.25	0.88 ± 0.01	11,348.65 ± 1,295.18	95.80 ± 35.94	72.00 ± 0.00	12.65 ± 0.01	571.55 ± 125.23	62.72 ± 7.92

B(H3.1.1)	*Yarrowia lipolytica*	5.96 ± 0.31	1.64 ± 0.41	50.13 ± 6.37	0.87 ± 0.05	9,651.26 ± 578.15	91.89 ± 35.94	50.13 ± 1.94	12.45 ± 1.77	650.90 ± 325.27	58.31 ± 16.73
A(E3)		6.31 ± 0.73	1.82 ± 0.08	58.84 ± 7.06	0.96 ± 0.01	11,784.52 ± 165.79	89.93 ± 11.06	62.25 ± 9.90	13.08 ± 1.39	818.80 ± 234.19	66.80 ± 6.02
C(E2.1)	*Meyerozyma caribbica*	5.69 ± 0.09	1.58 ± 0.52	49.82 ± 4.12	0.87 ± 0.01	10,742.44 ± 656.09	87.98 ± 13.82	67.75 ± 0.35	14.94 ± 1.73	556.60 ± 68.31	63.57 ± 2.25
A(F8.2)		5.95 ± 0.01	1.16 ± 0.24	51.20 ± 0.88	0.90 ± 0.04	10,800.56 ± 1,424.13	78.20± 0.00	54.38 ± 0.53	13.30 ± 0.88	557.75 ± 4.88	65.69 ± 6.75
C(H5.1)	*Wickerhamomyces anomalus*	5.80 ± 0.08	1.76 ± 0.01	59.16 ± 0.72	0.94 ± 0.13	11,113.18 ± 1,370.28	64.52 ± 2.76	51.50 ± 0.00	12.51 ± 1.54	678.50 ± 237.45	60.85 ± 11.70
C(F10.4)	*Pichia kluyveri*	5.43 ± 0.40	2.42 ± 0.65	56.28 ± 3.75	1.06 ± 0.00	12,151.25 ± 1,024.52	220.92 ± 135.47	67.75 ± 10.96	13.43 ± 0.47	817.65 ± 190.28	74.83 ± 14.01
C(G9)		5.84 ± 0.15	1.23 ± 0.07	50.18 ± 1.39	0.99 ± 0.02	12,295.54 ± 86.44	82.11 ± 0.00	64.75 ± 4.24	12.92 ± 0.37	655.50 ± 22.77	68.73 ± 1.57
C(A11.2)	*Metschnikowia pulcherrima*	5.66 ± 0.04	1.44 ± 0.03	53.28 ± 0.21	0.88 ± 0.13	10,204.37 ± 1,941.35	107.53 ± 8.29	65.75 ± 17.68	11.27 ± 0.75	694.60 ± 16.26	56.63 ± 13.19
B(B5)		5.63 ± 0.28	1.58 ± 0.04	47.03 ± 1.12	0.84 ± 0.00	9,412.79 ± 280.57	111.44 ± 13.82	51.50 ± 17.68	11.80 ± 0.59	765.90 ± 315.51	62.24 ± 6.19
A(F2.1)	*Rhodotorula mucilaginosa*	5.45 ± 0.03	1.93 ± 0.09	53.64 ± 10.73	1.03 ± 0.00	11,084.12 ± 181.38	99.71 ± 19.35	53.00 ± 4.95	13.88 ± 1.81	825.70 ± 39.03	82.84 ± 15.10
Control	Untreated	5.48 ± 0.01	1.28 ± 0.16	52.91 ± 2.43	0.91 ± 0.05	13,547.04 ± 5,211.88	111.44 ± 8.29	51.75 ± 5.66	13.18 ± 0.40	817.65 ± 316.38	68.68 ± 14.51
